# The impact of genomic distance on enhancer‐promoter interactions at the *CFTR* locus

**DOI:** 10.1111/jcmm.18142

**Published:** 2024-02-19

**Authors:** Jenny L. Kerschner, Frederick Meckler, Giuliana C. Coatti, Nirbhayaditya Vaghela, Alekh Paranjapye, Ann Harris

**Affiliations:** ^1^ Department of Genetics and Genome Sciences Case Western Reserve University Cleveland Ohio USA; ^2^ Present address: Department of Genetics University of Pennsylvania Philadelphia Pennsylvania USA

**Keywords:** 4C‐seq, chromatin structure, gene editing therapeutics, gene expression, promoter interactions

## Abstract

We identified and characterized multiple cell‐type selective enhancers of the *CFTR* gene promoter in previous work and demonstrated active looping of these elements to the promoter. Here we address the impact of genomic spacing on these enhancer:promoter interactions and on *CFTR* gene expression. Using CRISPR/Cas9, we generated clonal cell lines with deletions between the −35 kb airway enhancer and the *CFTR* promoter in the 16HBE14o^−^ airway cell line, or between the intron 1 (185 + 10 kb) intestinal enhancer and the promoter in the Caco2 intestinal cell line. The effect of these deletions on *CFTR* transcript abundance, as well as the 3D looping structure of the locus was investigated in triplicate clones of each modification. Our results indicate that both small and larger deletions upstream of the promoter can perturb *CFTR* expression and −35 kb enhancer:promoter interactions in the airway cells, though the larger deletions are more impactful. In contrast, the small intronic deletions have no effect on *CFTR* expression and intron 1 enhancer:promoter interactions in the intestinal cells, whereas larger deletions do. Clonal variation following a specific *CFTR* modification is a confounding factor particularly in 16HBE14o^−^ cells.

## 1 INTRODUCTION

Advances in nuclease‐based genome editing technologies have led to an increase in research into therapeutic gene editing, which has the potential to not only correct ‘rare’ monogenic diseases, but also common diseases with more complex genetic origins (reviewed in^1^, ^2^). Nucleases used for these purposes include zinc finger nucleases (ZFNs),^3^, ^4^ transcription activator‐like effector nucleases (TALENs), and CRISPR‐Cas9 related nucleases, which are able to induce double‐strand breaks (DSBs) at targeted sites in the genome. If a donor DNA template is provided, cells can repair the DSB using homology‐directed repair (HDR), otherwise, random indels may be incorporated at break sites through use of non‐homologous end joining (NHEJ). Large deletions and insertions can be induced when two neighbouring sites are targeted simultaneously. Engineered nucleases, such as deaminase‐linked Cas9 nickases can be used during base editing to chemically alter bases and thus directly change the identity of a nucleic acid, however there are many sequence‐dependent constraints that limit the broad‐application of this technique.^5^, ^6^ Genome editing using site‐specific nucleases can be applied in various ways for the development of therapies for monogenic diseases (reviewed in^1^, ^2^, ^7^), including by correction of pathogenic sequence variants or through gene inactivation. However, these strategies are often more applicable to diseases with few disease‐causing variants or mutation hotspots, as most affected individuals may benefit from an individual therapeutic. For monogenic disorders that can be caused by hundreds of disease‐causing variants, nuclease‐mediated gene therapies can also be used to introduce partial cDNAs either into endogenous or safe‐harbour loci.

Cystic fibrosis (CF) is one such monogenic disease in which nuclease‐mediated cDNA insertion is being investigated as a potential therapeutic. CF, a common autosomal recessive life‐limiting disorder, is caused by mutations in the cystic fibrosis transmembrane conductance regulator (*CFTR*) gene. CFTR is an ATP‐dependent chloride and bicarbonate ion channel that is required for normal epithelial fluid transport. The *CFTR* gene encompasses ~189 kb of genomic DNA, though the topologically associating domain (TAD) containing flanking regulatory elements for the *CFTR* locus spans ~316 kb (reviewed in^8^). More than 2000 variants are identified in *CFTR* (CFTR Mutation Database, http://www.genet.sickkids.on.ca), with 768 validated as disease‐causing or variants of clinical significance (https://cftr2.org, April 7, 2023 update), which are located within the promoter, exons, or splice sites. Disease‐causing variants are assigned to six classes depending on their impact on protein production, stability, or function.^9^ The fact that these *CFTR* variants occur across such a large genomic interval encourages efforts to develop a single gene‐based therapeutic to correct all of them in addition to CRISPR‐based prime editing techniques.^10^ To date several approaches to incorporate partial or complete copies of the *CFTR* cDNA into the endogenous *CFTR* locus or at a safe‐harbour locus have proven effective at restoration of functional CFTR in vitro.^11^, ^12^, ^13^, ^14^, ^15^, ^16^ Partial *CFTR* cDNAs/superexons have encompassed either exons 11–27 or 9–27 (RefSeq nomenclature) and were targeted to nearby introns or exons.^13^, ^16^ The complete *CFTR* cDNA was successfully inserted into exon 1 of the gene.^14^

One concern arising from the insertion of partial cDNAs into the *CFTR* locus for therapeutic purposes is whether these manipulations will disrupt the cell‐type selective or temporal regulation of the gene and impair gene expression levels. We and others have identified multiple intronic and extragenic *cis*‐regulatory elements (CREs) that confer cell‐selective expression on the gene.^17^, ^18^, ^19^, ^20^, ^21^ The TAD encompassing the *CFTR* locus is defined by ubiquitous sites of CCCTC‐binding factor (CTCF) occupancy at −80.1 kb upstream of the promoter and +48.9 kb downstream from the last exon. CTCF is an architectural protein with important roles in chromatin architecture and is a well characterized marker of TAD boundaries. Within this TAD the best studied CREs, and the most relevant to the current work, are upstream enhancers at −44 kb and −35 kb that are active in airway epithelial cells among other sites, and intronic enhancers in introns 1 and 11 (legacy nomenclature) that are utilized in intestinal epithelial cells and some other cell types.^22^, ^23^, ^24^, ^25^, ^26^, ^27^, ^28^, ^29^ These enhancers loop to the *CFTR* promoter to activate gene expression and their removal in the relevant cell types reduces *CFTR* expression, alters 3D organization of the entire genomic locus, and is often accompanied by changes in transcription factor (TF) recruitment or histone modifications at other *CFTR* CREs.^30^, ^31^, ^32^ A CTCF‐bound enhancer‐blocking insulator element, at −20.9 kb is important for *CFTR* regulation in both airway and intestinal cells.^33^, ^34^, ^35^, ^36^ Deletion of this element in 16HBE14o^−^ airway or Caco2 intestinal epithelial cell lines alters the normal CRE interaction profile^30^, ^31^ and causes a significant increase in *CFTR* expression in 16HBE14o^−^ cells, though the increase is not significant in Caco2 cells.

Here, with *CFTR* gene editing therapeutics as a focus, we specifically explore the constraints and flexibility of the *CFTR* locus to genomic alterations that impact the spacing between CREs and the gene promoter. Rather than deleting CREs or depleting activating TFs as in our previous work, we now manipulate intronic or extragenic regions with no known function to reduce the genomic intervals between critical enhancers and the gene promoter. This approach addresses the robustness of the locus to alterations in genomic spacing in the absence of potential secondary effects of inserted therapeutic partial cDNAs. We generated multiple clonal cell lines of *CFTR*‐expressing cells carrying CRISPR/Cas9‐mediated deletions of defined genomic fragments. Analysis of *CFTR* transcript abundance and higher order chromatin structure in these clones showed that expression of *CFTR* and locus organization are susceptible to disruption of the normal spacing between active CREs and the gene promoter.

## 2 MATERIALS AND METHODS

### 2.1 *CFTR* nomenclature

*CFTR* introns and exons are numbered using legacy nomenclature,^37^ for consistency with our previous body of work.^18^ Legacy to RefSeq conversion is published elsewhere.^32^

### 2.2 Cell culture

Human bronchial epithelial 16HBE14o^−^ ^38^ and human colon carcinoma Caco2^39^ cell lines were cultured in Dulbecco's modified Eagle's medium (low glucose), supplemented with 10% fetal bovine serum.

### 2.3 CRISPR‐mediated deletions

Pairs of gRNAs were identified to mediate the desired deletions using Benchling (benchling.com) and all had on‐target scores above 60 and off‐target scores above 40. Deletions were generated using gBlocks (see below) and a Cas9‐plasmid or ribonucleic protein (RNP) complexes. gBlocks containing the gRNA, expression sequences, and the gRNA scaffold (https://media.addgene.org/cms/filer_public/b9/08/b9081627‐6cd9‐43f3‐aa73‐f55b7a0fffaa/church_grna_cloning_protocol.pdf) were ordered from Integrated DNA Technologies, cloned into pSCB (Agilent), and sequence verified. 16HBE14o^−^ or Caco2 cells were forward transfected with a pair of pSCB‐gBlock plasmids and pMJ920 (encoding a WT GFP‐Cas9, Addgene #42234) (1:1:2 molar ratio) using Lipofectamine 3000 (ThermoFisher) following the manufacturer's protocol. 48‐h post transfection cells were harvested with Accutase (STEMCELL Technologies) for 16HBE14o^−^ and trypsin for Caco2 to release single cells. GFP+ cells were collected using fluorescence‐activated cell sorting (FACS) and manually plated into 96‐well plates for recovery of clones. RNPs complexes were made using Alt‐R™ S.p. Cas9‐GFP V3 (IDT) and gRNA consisting of a Alt‐R™ CRISPR‐Cas9 tracr‐RNA:crRNA duplex (IDT), following the manufacturer's protocol at a 1:1.2 Cas9‐GFP:gRNA ratio. Caco2 cells were reverse transfected with equal amounts of a pair of RNPs using Lipofectamine RNAiMAX (ThermoFisher) following the manufacturer's protocol. 20‐h post transfection, GFP+ cells were collected as above using FACS, and manually plated into 96‐well plates for recovery of clones. PCR of genomic DNA was performed to confirm the presence of homozygous deletions using multiple primer sets, and deletions confirmed by sequencing. For clones where there was allelic heterogeneity at the cut/repair sites surrounding the deletion, the deletion product was cloned into pSCA (Agilent), and plasmid clones were sequence verified to determine sequence of each deletion allele. gBlock and primer sequences are listed in Table S1.

### 2.4 Reverse transcription quantitative PCR (RT‐qPCR)

Total RNA was extracted from three serial passages of parental cells or deletion clones using TRIzol (Thermo Fisher, 15596018) following the manufacturer's protocol. cDNA was prepared using Taqman Reverse Transcription Reagents with random hexamers (Thermo Fisher, N8080234) and qPCR performed with Taqman Fast Advanced Master Mix (Thermo Fisher, 4444557). Primer and probe sequences are listed in Table S1. For all experiments with Caco2, cells were harvested for RNA extraction at 48 h post‐confluence, as *CFTR* expression increases post‐confluence.^40^ Multiple comparisons against WT cells after a Brown‐Forsythe and Welch one‐way ANOVA were used to determine statistical significance.

### 2.5 Western blot

For CFTR protein detection cells were lysed in NET buffer (10 mM Tris–HCl, pH 7.5, 150 mM NaCl, 5 mM EDTA, 1% [v/v] Triton X‐100, 1X Protease Inhibitor Cocktail [Sigma‐Aldrich, P8430]) as previously described^41^ and resolved by SDS‐PAGE using standard protocols on a 4% stacking and 7% resolving gels. Immobilon‐P PVDF membranes (Millipore‐Sigma, IPVH00010) were probed with primary antibodies specific for CFTR (Cystic Fibrosis Foundation, CFF‐596, lot # TJ20200121100285, 1:2000) and β‐tubulin (Sigma‐Aldrich, T4026, lot # 128M4790V, 1:20,000). The secondary antibody was anti‐mouse‐HRP (Agilent/Dako, P0447, lot # 20051789, 1:8000 or 1:10,000). Proteins were detected using ECL Western Blotting Substrate (Pierce).

### 2.6 4C‐seq

4C‐seq libraries were generated as previously described.^42^ Crosslinked chromatin from ~1 × 10^7^ cells was digested using NlaIII (primary) and DpnII or Csp6I (secondary) restriction enzymes. 4C experiments were performed at least twice from separate passages of each cell line, though only a single representative domainogram is shown in the results. Viewpoint primer sequences and enzyme pairs are shown in Table S1. Quantification of 4C‐seq reads were generated using the pipe4C pipeline v1.1 with default parameters.^42^ Read density tracks of replicates were merged and then subtracted from WT 16HBE14o^−^ or WT Caco2 using the deepTools bigwigCompare tools.^43^

## 3 RESULTS

In order to determine the capacity of the *CFTR* locus to respond to genomic disruptions, we measured the impact of reducing the spacing between cell‐selective enhancers and the *CFTR* promoter. Specifically, we deleted predicted non‐regulatory, extragenic or intronic DNA sequences of different sizes at two sites in the locus, from *CFTR*‐expressing cells. Two cell lineages, airway and intestinal epithelial cells were targeted and expression of CFTR transcript and protein measured in combination with higher order locus interactions.

### 3.1 Reducing the distance between an airway enhancer and the *CFTR* promoter impacts gene expression and chromatin organization in airway epithelial cells

#### 3.1.1 Deletions upstream of the *CFT* promoter variably alter *CFTR* expression in 16HBE14o^−^ cells

**FIGURE 1:**
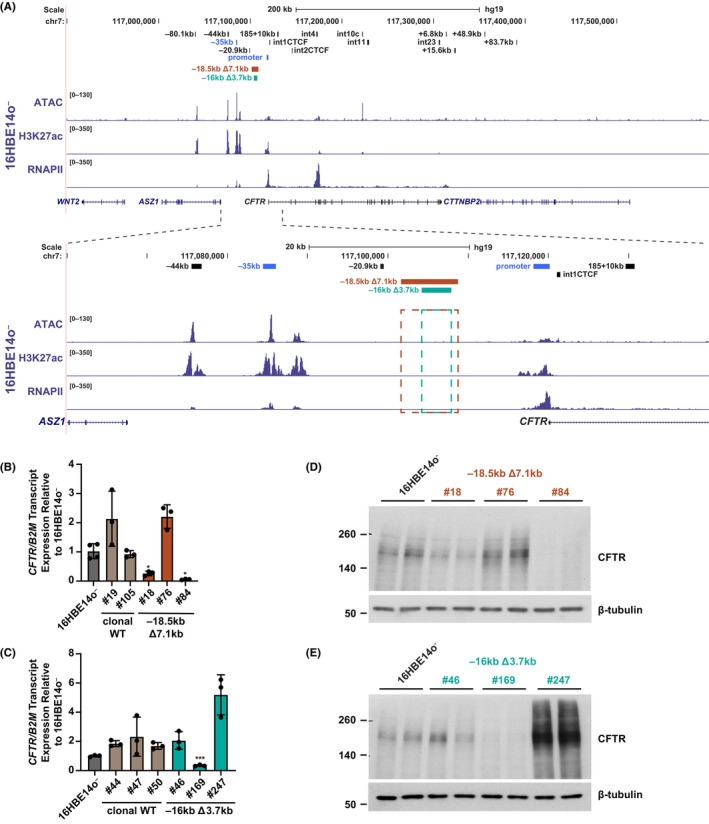
Deletions between upstream CREs and the promoter reduces *CFTR* expression in 16HBE14o^−^ cells. (A) Genomic coordinates of the *CFTR* locus are shown on hg19, with 16HBE14o^−^ open chromatin (by ATAC‐seq),^46^ and H3K27ac and RNAPII enrichment (ChIP‐seq)^31^ shown. Expanded area below details the sites targeted for deletion in 16HBE14o^−^ cells, with −18.5 kb Δ7.1 kb in brown and −16 kb Δ2.1 kb in teal. The *CFTR* promoter and −20.9 kb CRE flanking the deletion sites are shown in chalk blue. (B, C) *CFTR* transcript expression normalized to *B2M* is shown relative to 16HBE14o^−^ WT cells (*n* = 3 or 4). Clonal WT cells are shown with −18.5 kb Δ7.1 kb clones in (B), and −16 kb Δ3.7 kb clones in (C). Statistics: ***denotes *p* < 0.001 and *p < 0.05 using multiple comparisons against 16HBE14o^−^ WT cells after a Brown‐Forsythe and Welch one‐way ANOVA. (D, E) CFTR protein expression, with β‐tubulin as a loading control for −18.5 kb Δ7.1 kb clones (D) and −16 kb Δ3.7 kb clones (E).

First, in the airway epithelial line, 16HBE14o^−^, we generated two deletions that reduce the distance between the −35 kb enhancer and the *CFTR* promoter. The choice of location for these deletions was influenced by the presence of the CTCF‐bound insulator at −20.9 kb, which is known to be a key element in the 3D structure of the active *CFTR* locus and interacts directly with the −35 kb enhancer.^31^, ^44^ For this reason, we generated deletions 3′ to the −20.9 kb site: a 7.1 kb deletion with its 5′ end at −18.5 kb upstream of the *CFTR* translational start site (tss) (−18.5 kb Δ7.1 kb), and a smaller 3.7 kb deletion with its 5′ end at −16 kb upstream of the tss (−16 kb Δ3.7 kb), which falls within the larger 7.1 kb deletion. The targeted regions were chosen based upon a lack of open chromatin, H3K27ac enrichment, or RNAPII recruitment in 16HBE14o^−^ cells^25^ (Figure 1A), and thus are not predicted to have a regulatory function.

Two guide RNAs were designed for each deletion to induce CRISPR/Cas9‐mediated double strand breaks, and deletion of the intervening sequence. A minimum of three clones were isolated that were homozygous for each deletion based on PCR assays. The genotypes were verified by Sanger sequencing, which also determined the two Cas9 cut sites on each allele (Figure S1A,B). The expected target deletion was generated on both alleles of most clones (*n* = 2/6 for −18.5 kb Δ7.1 kb; *n* = 3/6 for −16 kb Δ3.7 kb), however, some alleles had slightly larger deletions (maximum Δ + 16 bp for −18.5 kb Δ7.1 kb; maximum Δ + 9 bp for −16 kb Δ3.7 kb). However, all deletions begin at the same location ~15.6 kb or 18.1 kb 3′ to the −35 kb enhancer (and hence ~2.2 kb or 17.4 kb downstream of the −20.9 kb insulator).

The effect on *CFTR* gene expression following deletion was variable in both the −18.5 kb Δ7.1 kb and −16 kb Δ3.7 kb deletion clones. Of note, clonal WT cells (which did not have deletions of either allele at the −18.5 kb and −16.5 kb sites) also showed some variation in *CFTR* expression compared to WT bulk 16HBE14o^−^ cells (~ 2‐fold in both series, Figure 1B,C), however this variation was not statistically significant. Notably, the Δ7.1 kb clones #18 and #84 had significantly less *CFTR* expression (25% and 6% of WT bulk 16HBE14o^−^ cells, respectively). Though the Δ7.1 kb clone #76 had ~2‐fold more *CFTR* mRNA compared to WT 16HBE14o^−^ (Figure 1B), this was within the range for non‐targeted WT cells and was not statistically significant. Next, measuring *CFTR* expression in the −16 kb Δ3.7 kb clones, none of the clonal WT cells had significant expression differences compared to WT 16HBE14o^−^ cells, nor did clone #46, in which expression levels were similar to the clonal WT cells. Clone #169 showed a significant decrease in expression to ~33% of WT 16HBE14o^−^. However, the ~2.5‐fold increase in *CFTR* expression over the clonal WT range and ~5‐fold increase over WT 16HBE14o^−^ cells observed in clone #247 was not statistically significant (Figure 1C). Western blot analysis of CFTR protein expression in these clones showed a close correlation with *CFTR* transcript abundance. Substantially reduced levels of CFTR were seen in clones #18 and #84 and higher amounts in clone #76 for the Δ7.1 kb clones (Figure 1D), while for the Δ3.7 kb clones #169 showed very low levels of protein, #247 very high levels and #46 showed passage dependent variation which was not greatly different from WT (Figure 1E). Together these data suggest that reducing the spacing between the −35 kb enhancer and the *CFTR* promoter can alter *CFTR*/CFTR expression levels in 16HBE14o^−^ cells. However, though the larger deletion had a more substantial effect on *CFTR*/CFTR expression (greater loss in two Δ7.1 kb clones compared to two Δ3.7 kb clones), clonal variation in this cell line makes quantitative interpretation challenging.

#### 3.1.2 The higher order chromatin structure of the *CFTR* locus is sensitive to genomic deletions in individual 16HBE14o^−^ clones

Next, to determine whether the changes in *CFTR* gene expression observed in the −18.5 kb Δ7.1 kb and −16 kb Δ3.7 kb deletion clones were associated with alterations in long‐range chromatin interactions at the locus, we preformed circular chromosome conformation capture followed by deep sequencing (4C‐seq). Of particular interest were the strong interactions between the −35 kb enhancer and the *CFTR* promoter that were observed in our previous analyses.^31^, ^44^ Based upon this earlier work, we assessed changes in the interaction profiles between bulk WT 16HBE14o^−^ cells and deletion clones using 4C‐seq viewpoints located at the 5′ TAD boundary (−80.1 kb), the −20.9 kb insulator, and the *CFTR* promoter.

**FIGURE 2:**
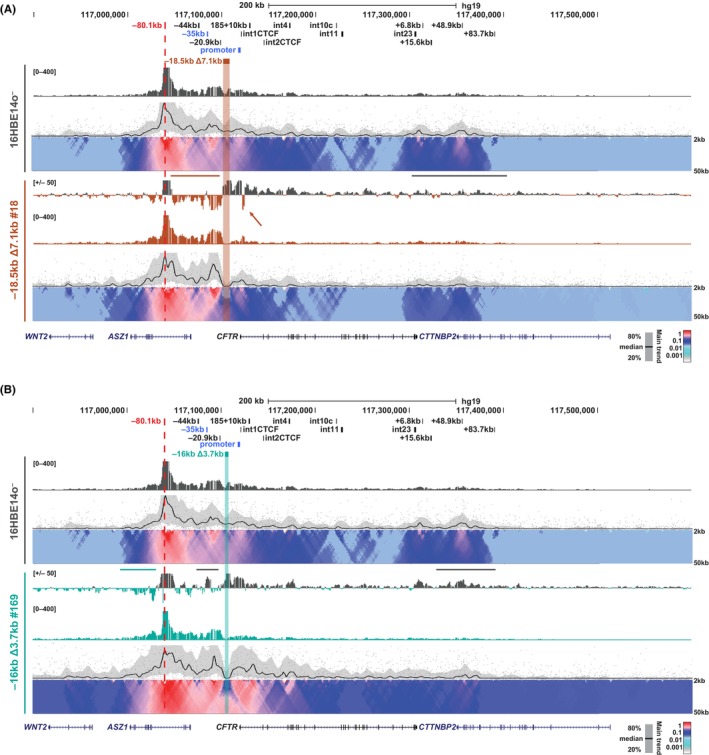
Impact of upstream deletions on interactions with the 5′ TAD boundary in 16HBE14o^−^ cells. 4C‐seq analysis of 16HBE14o^−^ WT (grey; A and B) and individual −18.5 kb Δ7.1 kb clones (brown; A) and −16 kb Δ3.7 kb clones (teal; B) with a viewpoint at the −80.1 kb 5′ TAD boundary (red dotted line). Key *CFTR* CREs and the deletion site are shown at the top. Read quantification tracks from an average of two replicates are shown for each cell type (grey or single coloured tracks) and a representative domainogram immediately below. Interaction profile subtraction tracks, for each deletion clone with respect to WT 16HBE14o^−^ are shown in log_2_ scale. Losses (above) and gains (below) in interactions from 16HBE14o^−^ WT are shown with respect to the y‐axis. Regions of interest are marked by horizontal bars or arrows. Data for additional clones are shown in Figures S2 and S3.

In WT 16HBE14o^−^ cells the 5′ and 3′ TAD (+48.9 kb) boundaries interact, in addition to the −80.1 kb boundary interacting with the airway enhancers at −44 kb and −35 kb, the −20.9 kb insulator, the *CFTR* promoter and an element in intron 4 that we reported previously^30^, ^32^ (Figure 2A, top domainogram). In −18.5 kb Δ7.1 kb clones #18 and #84 in which *CFTR* expression is reduced, the interactions between the 5′ TAD boundary and the region upstream of the deletion are strengthened (Figure 2A; Figure S2, brown lines and gain of interactions in brown below the line in the bigwig subtraction track). In contrast, few changes in interactions were seen across this region in clone #76 in which *CFTR* expression increased compared to WT 16HBE14o^−^ (Figure S2). Notably, in clone #18, which maintains 25% of *CFTR* levels compared to 6% in clone #84, a unique interaction is observed between the −80.1 kb viewpoint and a known site of CTCF occupancy in intron 1, (Figure 2A, brown arrow) and this is accompanied by a loss of interactions of regions flanking this CTCF site. All three Δ7.1 kb clones display reduced interactions between −80.1 kb and downstream sites spanning intron 23 (legacy) through to the 3′ TAD boundary at +48.9 kb, with the reductions most apparent in −18.5 kb Δ7.1 kb clones #76 and #84 (Figure 2A, Figure S2, grey lines). This reduction in interaction between −80.1 kb and the downstream region spanning intron 23 to +48.9 kb is also observed in the three small −16 kb Δ3.7 kb deletion clones, irrespective of the change in *CFTR* expression in these cells (Figure 2B; Figure S3, grey lines). Uniquely in −16 kb Δ3.7 kb clone #46, a gain in interactions is seen between −80.1 kb and enhancers at −44 kb and −35 kb (Figure S3, teal line), while in the other −16 kb Δ3.7 kb clones, these interactions become weaker compared to WT 16HBE14o^−^ cells (Figure 2B; Figure S3, grey lines). Also of note in clone #169, is the increase in interactions of the −80.1 kb viewpoint with regions 5′ to the TAD boundary (Figure 2B; Figure S3, teal line), which we have seen previously upon perturbations of the locus architecture.^31^ Together, these data indicate the interactions between the 5′ and 3′ TAD boundaries in 16HBE14o^−^ cells are easily perturbed by deletions generated between the −35 kb enhancer and the *CFTR* promoter.

**FIGURE 3:**
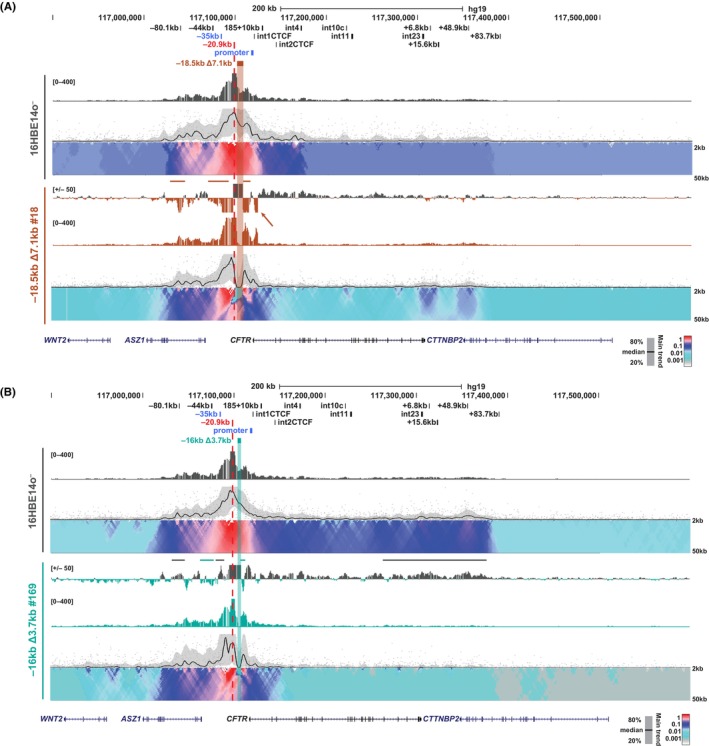
Impact of upstream deletions on interactions with the −20.9 kb insulator in 16HBE14o^−^ cells. 4C‐seq analysis of 16HBE14o^−^ WT (grey; A and B)^46^, ^47^ and individual −18.5 kb Δ7.1 kb clones (brown; A) and −16 kb Δ3.7 kb clones (teal; B) with the viewpoint at the −20.9 kb insulator (red dotted line). See Figure 2 legend for detailed description of tracks shown. Data for additional clones are shown in Figures S4 and S5.

We also examined the changes in interactions with a viewpoint at the −20.9 kb insulator in the 16HBE14o^−^ deletion clones. All deletion clones showed markedly increased interactions between −20.9 kb and the region downstream of the deletions, irrespective of their size (Δ7.1 kb or Δ3.7 kb) or the impact on *CFTR* expression (Figure 3A; Figure S4, brown lines; Figure 3B; Figure S5, teal lines). Both −18.5 kb Δ7.1 kb deletion clones with reduced *CFTR* expression (#18 and #84) have similar changes in interactions upstream of the −20.9 kb viewpoint, which include interaction gains with the airway enhancers at −44 kb and −35 kb, as well as with the 5′ TAD boundary at −80.1 kb (Figure 3A; Figure S4, brown lines). In contrast, the −18.5 kb Δ7.1 kb #76 clone, which has increased *CFTR* expression compared to WT 16HBE14o^−^, displayed a decrease in interactions between −20.9 kb and the upstream CREs (Figure S4, grey lines). Interestingly, in −18.5 kb Δ7.1 kb clone #18, a strong interaction was also seen between the −20.9 kb viewpoint and the intron 1 CTCF site, consistent with enhanced interactions between this site and the −80.1 kb 5′ TAD boundary observed in Figure 2A (Figure 3A brown arrow). In the −16 kb Δ3.7 kb clones compared to WT 16HBE14o^−^, changes in interactions with the −20.9 kb viewpoint, included decreases with the −35 kb enhancer, the −80.1 kb 5′ TAD boundary, and sites spanning from the middle of the locus through to the 3′ TAD boundary at +48.9 kb (Figure 3B; Figure S5, grey lines). Uniquely, −16 kb Δ3.7 kb clone #169, which is the only small deletion clone with decreased *CFTR* expression, shows increased interactions between the −44 kb enhancer and the −20.9 kb viewpoint (Figure 3B, teal line). In general, and irrespective of changes to *CFTR* transcript levels, interaction profiles with the −20.9 kb viewpoint show a loss of interactions throughout the locus in the small deletion clones, reflecting a relaxation of the higher order structure of the *CFTR* locus, which is highly dependent on the −20.9 kb insulator element.^30^, ^31^ In contrast, in the large deletion clones this viewpoint tends to lose interactions downstream of the deletion and gain interactions upstream of the deletion in cells with reduced *CFTR* expression.

**FIGURE 4:**
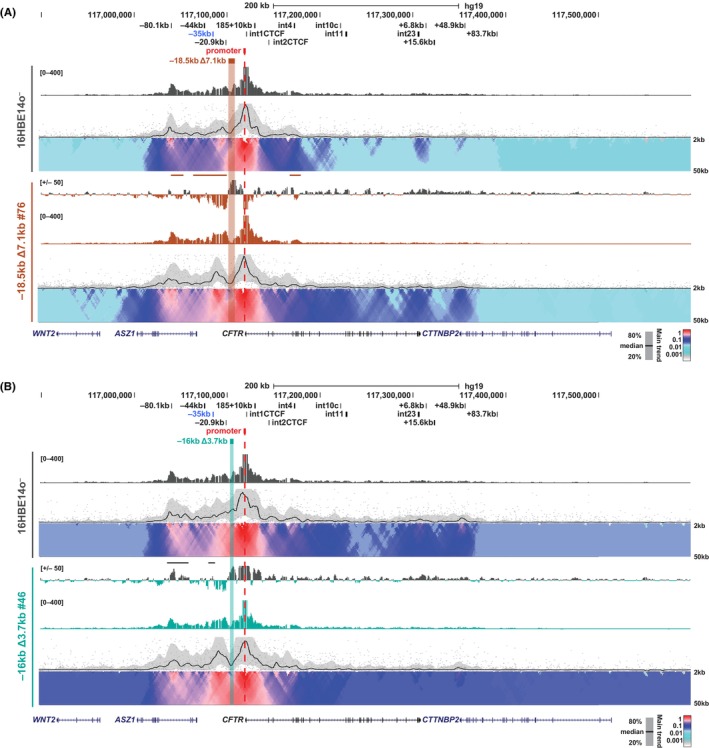
Impact of upstream deletions on interactions with the *CFTR* promoter in 16HBE14o^−^ cells. 4C‐seq analysis of 16HBE14o^−^ WT (grey; A and B) and individual −18.5 kb Δ7.1 kb clones (brown; A) and −16 kb Δ3.7 kb clones (teal; B) with the viewpoint at the *CFTR* promoter (red dotted line). See Figure 2 legend for detailed description of tracks shown. Data for additional clones are shown in Figures S6 and S7.

Lastly, using the *CFTR* promoter as a viewpoint, predominant interactions are with the −80.1 kb 5′ TAD boundary, the −44 kb and −35 kb enhancers, and the −20.9 kb insulator in WT 16HBE14o^−^ cells (Figure 4A, top domainogram). All but one (−18.5 kb Δ7.1 kb #18) of the deletion clones (both Δ7.1 and Δ3.7 kb) show an increased interaction between the *CFTR* promoter and −20.9 kb (Figure 4A; Figure S6, brown lines; Figure 4B; Figure S7, teal lines), which is reciprocal to the increased interactions observed between the −20.9 kb viewpoint and sites immediately downstream of the deletions. In the −18.5 kb Δ7.1 kb deletions, all clones show increased interactions between the promoter viewpoint and intron 4, whereas clones #76 and #84 have similar gains in interactions between the promoter viewpoint and sites upstream of the deletion (−80.1 kb, −44 kb and −35 kb), while clone #18 has decreased interactions over this region (Figure 4A; Figure S6, brown and grey lines). In general, the changes in interaction profiles of the −16 kb Δ3.7 kb clones are similar among the three clones, which show decreased interactions between the promoter and −80.1 kb and the −35 kb enhancer (Figure 4B; Figure S7, grey lines). Together these data indicate the normal interactions the *CFTR* promoter with *CFTR* CREs is highly susceptible to alterations in spacing between the −35 kb enhancer and the promoter.

### 3.2 Reducing the distance between an intronic enhancer and the *CFTR* promoter has a modest effect on gene expression and chromatin organization in intestinal cells

#### 3.2.1 Intronic deletions have a size‐dependent impact on *CFTR* expression in Caco2 cells

**FIGURE 5:**
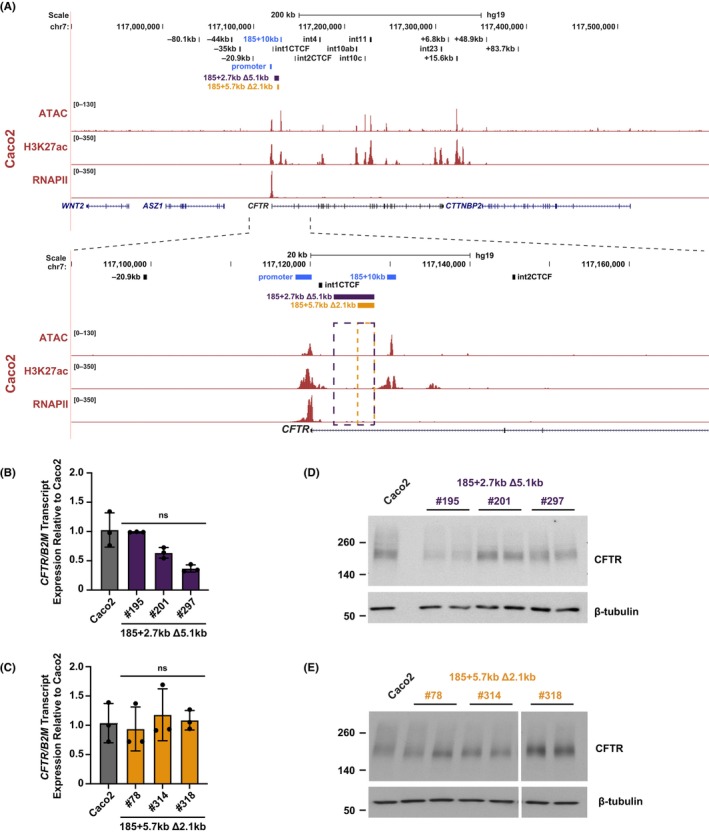
Deletions between the *CFTR* promoter and an intestinal intronic enhancer have little impact on *CFTR* expression in Caco2 cells. (A) Genomic coordinates of the *CFTR* locus are shown on hg19, with Caco2 open chromatin (by ATAC‐seq),^45^ and H3K27ac and RNAPII enrichment (ChIP‐seq)^31^ shown. Expanded area shown below details the sites targeted for deletion in Caco2 cells, with 185 + 2.7 kb Δ5.1 kb in purple and 185 + 5.7 kb Δ2.1 kb in gold. The *CFTR* promoter and intron 1 CRE flanking the deletion sites are shown in chalk blue. (B, C) *CFTR* transcript expression normalized to *B2M* is shown relative to Caco2 WT cells (*n* = 3) is shown for 185 + 2.7 kb Δ5.1 kb clones in (B) and 185 + 5.7 kb Δ2.1 kb clones in (C). Statistics: ns denotes not significant p‐value using multiple comparisons against Caco2 WT cells after a Brown‐Forsythe and Welch one‐way ANOVA. (D, E) CFTR protein expression, with β‐tubulin as a loading control for 185 + 2.7 kb Δ5.1 kb clones (D) and 185 + 5.7 kb Δ2.1 kb clones (E).

In contrast to 16HBE14o^−^ cells in which *CFTR* expression is regulated primarily by upstream enhancers at −44 kb and −35 kb (Figure 1A), in intestinal cells, both in primary colon organoids^45^ and the Caco2 intestinal cell line, the gene is controlled by intronic enhancers. The dominant enhancers include a relatively weak one in intron 1 (185 + 10 kb),^19^, ^20^, ^41^ which works together with a much stronger one in intron 11 (1811 + 0.8 kb, RefSeq intron 12).^27^, ^28^, ^32^ To investigate the effect of spacing on the intron 1 enhancer:promoter interactions, we next generated clones of Caco2 cells carrying one of two deletions between the *CFTR* promoter and this enhancer (Figure 5A). Importantly the deletions, a 5.1 kb deletion at 185 + 2.7 kb and a 2.1 kb deletion at 185 + 5.7 kb (which share the same 3′ cut site), do not impinge upon a known site of CTCF occupancy upstream of the intron 1 enhancer (185 + 1 kb). As for the genomic regions deleted in 16HBE14o^−^ cells, these intronic regions lack open chromatin, H3K27ac and RNAPII enrichment in Caco2 cells (Figure 5A) and are predicted to not have regulatory function.

Three clones of each Caco2 deletion were generated and verified using Sanger sequencing (Figure S8). As observed in our previous CRISPR/Cas9 deletion series in Caco2 cells,^30^, ^32^ all the clones selected for further analysis were homozygous for the expected deletion (note: Caco2 is triploid for *CFTR*), though there was minor variation in the sequence at the cut sites. While all clones had the expected deletion on the majority of alleles, 185 + 5.7 kb Δ2.1 kb clone #78 and 185 + 2.7 kb Δ5.1 kb clones #201 and #297 also contained unique alleles with either additional upstream deletions, complex insertions at the deletion site or extended deletions which are detailed in Figure S8. Notably no WT alleles were detected in these clones based upon PCR of genomic DNA.

The impact of the deletions on *CFTR* expression was measured by RT‐qPCR and compared to WT bulk Caco2 cells (Figure 5B, C), since our previous extensive experiments in Caco2 showed very little WT clonal variation in this line^30^, ^32^ The 185 + 5.7 kb Δ2.1 kb deletion had a very minor and not significant effect on *CFTR* expression in the three Caco2 deletion clones analysed (#78, #314, #318, Figure 5C). The larger deletion had a variable effect on *CFTR* expression in the 185 + 2.7 kb Δ5.1 kb clones #195, #201 and #297. There was a comparative reduction of 36%–63% in two of the three clones with the deletion, however this did not reach statistical significance (Figure 5B). Little change was detected in CFTR protein levels in cells carrying either the large or small deletions and moreover there was no direct correlation with *CFTR* mRNA levels in each clone (Figure 5D,E). Of note the RNA and protein was not collected in parallel from each clone and since *CFTR* expression is particularly sensitive to minor variations in confluence in Caco2 cells, this may have bearing on these results. However, all protein lysates were extracted at 48 h post direct observation of confluence, as for the RNA collections.

#### 3.2.2 Unique changes to the CFTR locus interaction profile in individual Caco2 deletion clones

We next performed 4C‐seq to investigate potential changes to the long‐range interactions across the *CFTR* locus in the Caco2 deletion clones. Viewpoints used included those at −80.1 kb, −20.9 kb and the *CFTR* promoter, as for the 16HBE14o^−^ clones, and additional ones in intron 1 distal to the targeted deletions (at 185 + 19.5 kb) and at the +48.9 kb 3′ TAD boundary. Fewer changes were observed in the 3D organization of *CFTR* in the context of the 185 + 5.7 kb Δ2.1 kb and 185 + 2.7 kb Δ5.1 kb deletions in Caco2 cells, compared to the deletions in 16HBE14o^−^ cells. First, with a viewpoint at the −80.1 kb 5′ TAD boundary no consistent change in looping was detected in any of the Caco2 deletion clones, irrespective of deletion size (Figure S9). With the promoter viewpoint, several deletion clones (185 + 2.7 kb Δ5.1 kb #297 and 185 + 5.7 kb Δ2.1 kb #314 and #318) showed a small gain in interactions with the −80.1 kb site (Figure S10, arrow), though this did not correlate with any changes in gene expression, perhaps implicating a compensatory mechanism to stabilize the TAD after genomic deletions.

**FIGURE 6:**
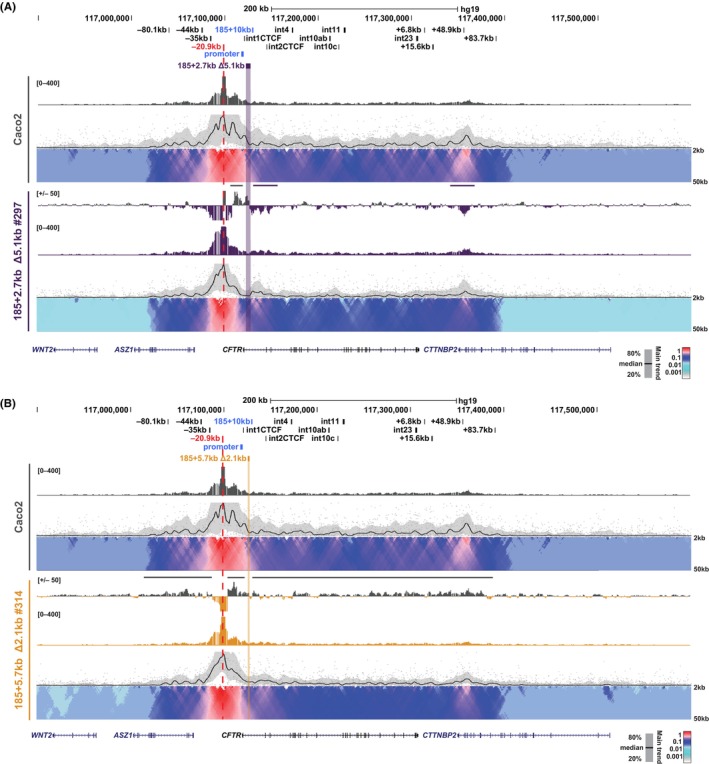
Impact of intronic deletions on interactions with the −20.9 kb CRE in Caco2 cells. 4C‐seq analysis of Caco2 WT (grey; A and B) and individual 185 + 2.7 kb Δ5.1 kb clones (purple; A) or 185 + 5.7 kb Δ2.1 kb clones (gold; B) with a viewpoint at the −20.9 kb CRE (red dotted line). Key *CFTR* CREs as well as the deletion are shown at the top. Read quantification tracks from an average of two replicates are shown for each cell type (grey or single coloured tracks) and a representative domainogram immediately below. Interaction profile subtraction tracks, for each deletion clone with respect to WT Caco2 are shown in log_2_ scale. Losses (above) and gains (below) in interactions from Caco2 WT are shown with respect to the y‐axis. Regions of interest are marked by horizontal bars or arrows. Data for additional clones are shown in Figures S11 and S12.

Using a viewpoint at the −20.9 kb site, most of the Caco2 deletion clones showed similar changes in interaction profiles compared to the WT locus. The 185 + 2.7 kb Δ5.1 kb clones #195 and #201 and all three 185 + 5.7 kb Δ2.1 kb clones show a general loss of interactions between the −20.9 kb viewpoint and the entire locus 3′ of the viewpoint (Figure 6; Figures S11 and S12, grey lines). Most of these clones also display a loss of interactions 5′ to the viewpoint that encompasses sequences extending beyond the 5′ TAD boundary at −80.1 kb (Figure 6; Figures S11 and S12, grey lines), with the exception of 185 + 5.7 kb Δ2.1 kb #78, which has a more complex genotype (Figure S8B). Clone 185 + 2.7 kb Δ5.1 kb #297, which has a larger (5.4 kb instead of 5.1 kb) deletion on one allele, has a different interaction profile than the other clones, with notable gains in interactions between the viewpoint and the region immediately downstream of the deletion and with the +48.9 kb 3′ TAD boundary (Figure 6A; Figure S11, purple lines). Both the large and small deletion clones show a loss of interactions between the −20.9 kb viewpoint and a region 5′ to the deletion, which includes the *CFTR* promoter (Figure 6; Figures S11 and S12, grey lines). These data are consistent with our earlier observations that although removal of the −20.9 kb insulator had a dramatic effect on higher order chromatin structure at the *CFTR* locus in Caco2 cells, it had little impact on *CFTR* gene expression.^30^

**FIGURE 7:**
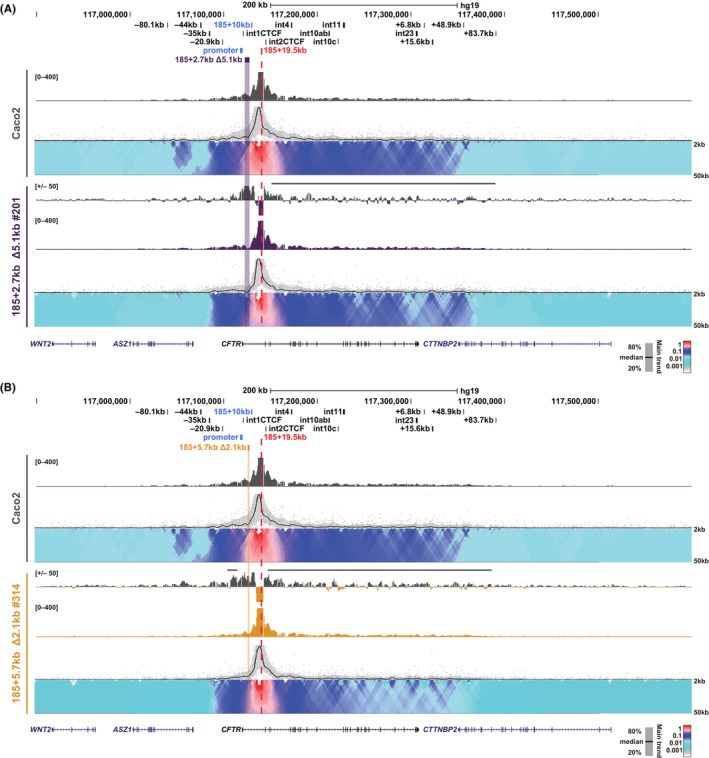
Impact of intronic deletions on interactions with *CFTR* intron 1 (185 + 19.5 kb) in Caco2 cells. 4C‐seq analysis of Caco2 WT (grey; A and B) and individual 185 + 2.7 kb Δ5.1 kb clones (purple; A) or 185 + 5.7 kb Δ2.1 kb clones (gold; B) with the viewpoint at intron 1 (185 + 19.5 kb) of *CFTR* (red dotted line). See Figure 6 legend for a detailed description of tracks shown. Data for additional clones are shown in Figure S13 and S14.

Changes to interactions with the intron 1 (185 + 19.5 kb) viewpoint are similar to those seen with the −20.9 kb viewpoint, with a relative loss of interactions across the whole locus 3′ of the viewpoint in all clones except 185 + 2.7 kb Δ5.1 kb clone #297, which has a more complex profile of gains and losses across the region. (Figure 7; Figures S13 and S14, grey lines). Similarly, with this viewpoint there is a general loss of interactions upstream of the deletion, which is particularly extended in 185 + 2.7 kb Δ5.1 kb #297 and 185 + 5.7 kb Δ2.1 kb #314 (Figure 7; Figures S13 and S14, grey line).

**FIGURE 8:**
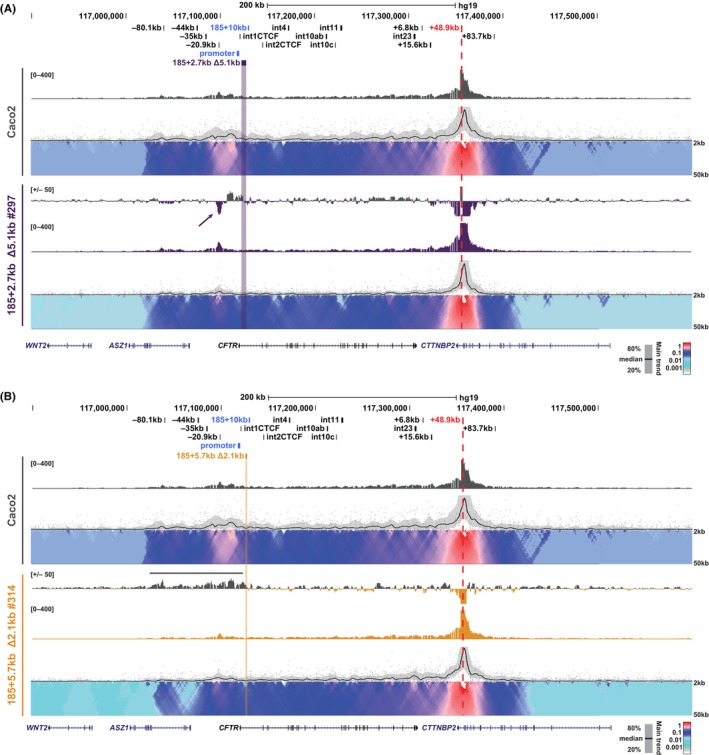
Impact of intronic deletions on interactions with the 3′ TAD boundary in Caco2 cells. 4C‐seq analysis of Caco2 WT (grey; A and B) and individual 185 + 2.7 kb Δ5.1 kb clones (purple; A) or 185 + 5.7 kb Δ2.1 kb clones (gold; B) with the viewpoint at the 3′ TAD boundary at +48.9 kb (red dotted line). See Figure 6 legend for detailed description of tracks shown. Data for additional clones are shown in Figure S15 and S16.

In WT Caco2 cells, the +48.9 kb viewpoint, interacts with the 5′ TAD boundary at −80.1 kb, and regions between the promoter and ~ −30 kb (Figure 8, top domainogram). In 185 + 2.7 kb Δ5.1 kb clones #195 and #201 there is a generalized loss of interactions with +48.9 kb across the locus, which are particularly evident in the region spanning −20.9 kb to the *CFTR* promoter (Figure S15, grey lines). In clone 185 + 2.7 kb Δ5.1 kb #297 an additional unusual feature of the interactions with the +48.9 kb viewpoint is a marked increase in interactions specifically at the −20.9 kb insulator element (Figure 8A, purple arrow). Of note, a reciprocal interaction gain was seen with the −20.9 kb viewpoint in this clone (Figure 6A). In clones 185 + 5.7 kb Δ2.1 kb #314 and #318, losses in interactions between the +48.9 kb viewpoint and the 5′ TAD boundary are particularly evident, together with a general loss in interactions from this boundary through to the deletion (Figure 8B; Figure S16, grey lines). In 185 + 5.7 kb Δ2.1 kb clone #78, which has a more complex genotype than the others, these losses are less pronounced. As with the 185 + 2.7 kb Δ5.1 kb clones, the 185 + 5.7 kb Δ2.1 kb clones also show a generalized loss of interactions between +48.9 kb and the whole length of the *CFTR* locus (Figure 8B; Figure S16). Together these results suggest that while there are some general losses in interactions across the *CFTR* locus in both 185 + 2.7 kb Δ5.1 kb and 185 + 5.7 kb Δ2.1 kb clones, little change is seen in enhancer:promoter interactions, consistent with the minimal changes in *CFTR* expression.

## 4 DISCUSSION

We previously explored *CFTR* regulatory mechanisms in intestinal and airway cell lines by multiple approaches including depletion or ablation of TFs,^23^, ^24^, ^28^, ^29^, ^36^, ^44^ genomic deletion of CREs,^30^, ^31^, ^32^ and enhancer relocation.^46^ Importantly, we showed that most of the chromatin profile signatures of CREs that are identified in cell lines are also seen in the primary cells from the same lineages.^31^, ^45^ However, it is difficult to use primary cells for generating clonal isolates. In an effort to extend our understanding of normal *CFTR* regulation, which has implications for the development of CF genetic therapies, we reduced the distance between the *CFTR* promoter and its cell type‐selective enhancers in two relevant cell lines. By generating deletions of two sizes in each location, we are also able to investigate if alterations in *CFTR* expression or genome organization are proportional to spacing. Our results indicate that the *CFTR* locus is susceptible to deletions of regions with no known regulatory or other function, in a location and size dependent manner. The proximity of the deletion to known features of the higher order chromatin structure may also be relevant.

*CFTR* expression in the intestinal cell line, Caco2, is regulated in part through the activity and recruitment of intronic enhancers in introns 1 (185 + 10 kb) and 11 (1811 + 0.8 kb) to the *CFTR* promoter. While the intron 1 enhancer is ~10 kb from the *CFTR* promoter, the intron 11 enhancer, which is the stronger of the two,^27^ is located ~100 kb from the *CFTR* promoter. The deletions we generated in Caco2 cells were 2.7 kb and 5.8 kb into intron 1 and were not expected to interfere with splicing of *CFTR* transcripts. While we previously showed that deletion of the intron 1 enhancer itself in Caco2 cells resulted in a 55% reduction in *CFTR* transcript levels,^32^ neither of the current deletions between this enhancer and the *CFTR* promoter caused significant changes in *CFTR* expression. Though two clones with the larger deletion in this region, 185 + 2.7 kb Δ5.1 kb clones #201 (*p* = 0.1372) and #297 (*p* = 0.0545) showed reduced *CFTR* expression, these values were not statistically significant (largely due to variation in the WT levels at different passages). Furthermore, both these clones have slightly larger (~100 and 300 bp, respectively) deletion on one of the three *CFTR* alleles, which may be a contributing factor. Of the Caco2 deletion clones, 185 + 2.7 kb Δ5.1 kb clone #297 displays both the greatest change in *CFTR* expression and the most unique changes in the interaction profile of the *CFTR* locus. Notably, many of the unique changes to the looping of *CFTR* in this clone are focused around sites known to bind CTCF, including the increased interaction of the promoter with −80.1 kb viewpoint (CTCF bound TAD boundary) (Figure S10), the increased interactions of the −20.9 kb CTCF‐bound insulator with the +48.9 kb 3′ TAD boundary (Figures 6A and 8A) and a region spanning the second half of intron 1 through to intron 3 (Figure 6A) which includes a known CTCF site in intron 2 of *CFTR*.^35^

Conversely, the clones generated in 16HBE14o^−^ cells, which have deletions between the −35 kb enhancer and the *CFTR* promoter, show much greater variation in both gene expression and looping of the *CFTR* locus. For this cell line the precise cut sites at each end of the deletion are very similar on each allele. The observed variability in the 16HBE14o^−^ clones could be due to a number of factors, including the identity of the original parental cell that incurred the deletion, proximity of the deletions to the CTCF site at the −20.9 kb insulator, or differences in the relative strengths of the −35 kb and intron 1 enhancers. We have recently reported on the heterogeneity of the 16HBE14o^−^ cell line, with single‐cell clonal isolates expressing up to a 1.9‐fold increase or 9.1‐fold decrease in *CFTR* transcript compared to bulk 16HBE14o^−^ cells.^47^ In light of these findings we examined and characterized multiple 16HBE14o^−^ non‐targeted and deletion clones. However, it is notable that we saw no correlation between the unique interaction profiles of the CFTR^high^ and CFTR^low^ cells previously characterized^47^ and the 16HBE14o^−^ deletion clones with increased or decreased *CFTR* expression compared to WT 16HBE14o^−^ (Figure 3; Figures S4 and S5). While intrinsic heterogeneity of the 16HBE14o^−^ cell line may contribute to the clonal variation in the impact of specific deletions in our results, it is likely not the main factor.

Some of the variation in *CFTR* expression within either large (7.1 kb) or small (3.7 kb) deletion clones of 16HBE14o^−^ is consistent with observed changes in interactions between CTCF‐bound structural elements and the 5′ airway enhancers, which is evident as alterations in the chromatin looping profiles. Notably, the clones in which *CFTR* expression was reduced (−18.5 kb Δ7.1 kb clones #18 and #84 and −16 kb Δ3.7 kb clone #169), showed increased interactions between CTCF sites at the 5′ TAD boundary, the −20.9 kb insulator, and one or both of the airway enhancers at −44 kb and −35 kb. These data are consistent with our earlier observations on KLF5‐null 16HBE14o^−^ cells in which *CFTR* expression is increased and there is a concurrent reduction in interactions between the 5′ TAD boundary, the −20.9 kb insulator, and the enhancers at −44 kb and −35 kb.^44^ Interestingly in the only −18.5 kb Δ7.1 kb clone with increased *CFTR* expression (clone #76), a decrease in interactions was observed between the −20.9 kb insulator and the upstream enhancers (Figure S4), while there were increased interactions between the promoter and enhancers (Figure S6) in this clone. In contrast, while 18.5 kb Δ7.1 kb clone #84 also shows increased interactions between the promoter and enhancers (Figure S6), it also has stronger interactions between the −20.9 kb insulator and these sites, which could contribute to the reduced *CFTR* expression in this clone. Additionally, in −18.5 kb Δ7.1 kb clone #84 a unique interaction occurs between the −80.1 kb and −20.9 kb CTCF sites and a previously characterized CTCF site in intron 1. Moreover, the loss of interactions of the promoter with all upstream CREs only in the 16HBE14o^−^ −18.5 kb Δ7.1 kb clone #18 may explain the reduced *CFTR* expression in this clone. Overall, the proximity of the 16HBE14o^−^ deletions to the −20.9 kb CTCF‐bound insulator might impair with the normal CTCF‐mediated interactions in cells.

Lastly, it is pertinent to compare the effects of the extragenic and intronic deletions on *CFTR* expression and looping, particularly with respect to the relative strength of the nearby enhancers, and the function of CTCF in maintaining enhancer:promoter interactions. The −35 kb CRE is a strong enhancer in 16HBE14o^−^ cells,^22^, ^23^ while the intron 11 CRE is a correspondingly strong enhancer in Caco2 cells.^27^ Deletion of these enhancers in the relevant cell types leads to nearly complete loss or 80% reduction in *CFTR* expression, respectively.^30^, ^31^ Conversely, only a 55% reduction in expression was observed in Caco2 cells following deletion of the intron 1 enhancer,^32^ which has less enhancer activity when compared to the intron 11 element.^26^, ^48^ As we see little change in the interactions between the *CFTR* promoter and sites along the locus in the Caco2 intronic deletion clones (Figure S10), it is likely that this small change in size of intron 1 has little effect on the looping of the intron 11 enhancer to the gene promoter.^30^ Conversely, as the extragenic enhancer at −35 kb is a relatively strong element that is closer to the promoter than the intron 11 CRE, it might be more susceptible to spacing perturbations. Additionally, it has been shown that promoter‐proximal CTCF sites (within 10 kb) are important for maintaining interactions between promoters and distal enhancers, whereas promoter‐proximal enhancers rely less on CTCF and cohesin for their activity.^49^, ^50^ As the intron 1 CTCF site (185 + 1 kb), a promoter‐proximal site, is unperturbed in the Caco2 deletions, this could also help explain the little impact of these deletions on *CFTR* expression and looping. Thus, reduction of spacing between the *CFTR* promoter and extragenic or intronic enhancers influences *CFTR* regulation and chromatin dynamics in a position‐dependent manner, that is likely influenced by neighbouring regulatory factors. Together these data provide further insight into the capacity of the *CFTR* locus to respond to non‐coding sequence disruptions and offer some guidance on approaches to target the *CFTR* locus for gene‐editing therapeutics, including relative strengths of any nearby regulatory elements and proximity to sites of occupancy of architectural proteins. It may be advantageous to evaluate chromatin dynamics and potential regulatory element disruption prior to embarking on *CFTR* partial cDNA‐mediated therapeutic protocols.

## AUTHOR CONTRIBUTIONS

**Jenny L. Kerschner:** Conceptualization (supporting); data curation (lead); formal analysis (equal); investigation (lead); methodology (equal); project administration (supporting); supervision (supporting); validation (equal); visualization (lead); writing – original draft (lead); writing – review and editing (equal). **Frederick Meckler:** Data curation (equal); formal analysis (equal); investigation (equal); methodology (supporting); writing – review and editing (supporting). **Giuliana C. Coatti:** Formal analysis (supporting); software (supporting); visualization (supporting); writing – review and editing (supporting). **Nirbhayaditya Vaghela:** Data curation (supporting); investigation (supporting); visualization (supporting); writing – review and editing (supporting). **Alekh Paranjapye:** Data curation (equal); formal analysis (equal); investigation (equal); methodology (equal); software (lead); validation (equal). **Ann Harris:** Conceptualization (lead); data curation (supporting); formal analysis (equal); funding acquisition (lead); investigation (supporting); methodology (supporting); project administration (lead); resources (equal); software (supporting); supervision (lead); validation (equal); visualization (supporting); writing – original draft (supporting); writing – review and editing (equal).

## FUNDING INFORMATION

This work was supported by the Cystic Fibrosis Foundation (Davis 19XX0, Harris 23XX0).

## CONFLICT OF INTEREST STATEMENT

The authors declare no conflicts of interest.

## Supporting information


Appendix S1.

## Data Availability

Data are deposited at NCBI GEO accession GSE239930.
