# Supplementary material for: The impact of genomic distance on enhancer‐promoter interactions at the CFTR locus
Source: J Cell Mol Med. 2024 Feb 19;28(4):e18142. doi: 10.1111/jcmm.18142 (PMC10875976; doi:10.1111/jcmm.18142)
Supplement: Supplementary file 1 — Appendix S1. [file JCMM-28-e18142-s001.pdf]

The impact of genomic distance on enhancer-promoter interactions at the *CFTR* locus.

Jenny L. Kerschner, Frederick Meckler, Giuliana C. Coatti, Nirbhayaditya Vaghela,

Alekh Paranjapye, and Ann Harris

Data Supplement

Figure Legends

Figures S1-S16

Table S1: Oligonucleotides

Supplementary Figure Legends

Figure S1: Sequence alignments of 16HBE14o$^-$ deletion clones. Sanger sequence alignments of deletion targeted areas are shown for 16HBE14o$^-$ -18.5kb Δ7.1kb clones **(A)** or -16kb Δ3.7kb clones **(B)**. Location and sequence of gRNAs is also shown. PCR amplicons were sequenced directly from genomic DNA, or from amplicons that were cloned into pSCA to distinguish multiple alleles. The allele deletion size(s) in each clone are also shown.

Figure S2: Extended analysis of interactions of the -18.5kb Δ7.1kb deletion clones with the 5' TAD boundary in 16HBE14o$^-$ cells. 4C-seq analysis of 16HBE14o$^-$ WT (grey) and -18.5kb Δ7.1kb clones (brown) with the viewpoint at the -80.1kb 5' TAD boundary (red dotted line). Key *CFTR* CREs as well as the deletion are shown at the top. Read quantification tracks from an average of two replicates are shown for each cell type (grey or single colored tracks) and a representative domainogram immediately below. Interaction profile subtraction tracks, for each deletion clone with respect to WT 16HBE14o$^-$ are shown in $\log_2$ scale. Losses (above) and gains (below) in interactions from 16HBE14o$^-$ WT are shown with respect to the y-axis. Regions of interest are marked by horizontal bars or arrows.

Figure S3: Extended analysis of the interactions of the -16kb Δ3.7kb deletion clones with the 5' TAD boundary in 16HBE14o$^-$ cells. 4C-seq analysis of 16HBE14o$^-$ WT (grey) and -16kb Δ3.7kb clones (teal) with the viewpoint at the -80.1kb 5' TAD boundary (red dotted line). See Figure S2 legend for detailed description of tracks shown.

Figure S4: Extended analysis of the interactions of the -18.5kb Δ7.1kb deletion clones with the -20.9kb insulator in 16HBE14o$^-$ cells. 4C-seq analysis of 16HBE14o$^-$ WT (grey) and -18.5kb Δ7.1kb clones (brown) with the viewpoint at the -20.9kb insulator (red dotted line). See Figure S2 legend for detailed description of tracks shown.

Figure S5: Extended analysis of the interactions of the -16kb Δ3.7kb deletion clones with the -20.9kb insulator in 16HBE14o- cells. 4C-seq analysis of 16HBE14o- WT (grey) or -16kb Δ3.7kb clones (teal) with the viewpoint at the -20.9kb insulator (red dotted line). See Figure S2 legend for detailed description of tracks shown.

Figure S6: Extended analysis of the interactions of the -18.5kb Δ7.1kb deletion clones with the *CFTR* promoter in 16HBE14o- cells. 4C-seq analysis of 16HBE14o- WT (grey) and -18.5kb Δ7.1kb clones (brown) with the viewpoint at the *CFTR* promoter (red dotted line). See Figure S2 legend for detailed description of tracks shown.

Figure S7: Extended analysis of the interactions of the -16kb Δ3.7kb deletion clones with the *CFTR* promoter in 16HBE14o- cells. 4C-seq analysis of 16HBE14o- WT (grey) and -16kb Δ3.7kb clones (teal) with the viewpoint at the *CFTR* promoter (red dotted line). See Figure S2 legend for detailed description of tracks shown.

Figure S8: Sequence alignments of Caco2 deletion clones. (A-B) Sanger sequence alignments of deletion targeted areas are shown for Caco2 185+2.7kb Δ5.1kb clones **(A)** or 185+5.7kb Δ2.1kb clones **(B)**. Location and sequence of gRNAs is also shown. Sequencing products were generated from PCR amplicons of genomic DNA, or from amplicons that were cloned into pSCA to distinguish multiple alleles. Deletion size of each clone or identified allele is also shown. Note that 185+5.7kb Δ2.1kb clone 78 has complex alleles with one allele having a secondary deletion upstream of the targeted deletion (allele 1) and another allele having a complex inverted sequence inserted upstream of the 5' cut site (blue letters), that contains an independent internal deletion (allele 4).

Figure S9: Impact of the 185+2.7kb Δ5.1kb and 185+5.7kb Δ2.1kb deletions on interactions with the 5' TAD boundary in Caco2 cells. 4C-seq analysis of Caco2 WT (grey), 185+2.7kb Δ5.1kb clones (purple), or 185+5.7kb Δ2.1kb clones (gold) with the viewpoint at the 5' TAD boundary at -80.1kb (red dotted line). Key *CFTR* CREs as well as the deletions are shown at the top and bottom. Read quantification tracks from an average

of two replicates is shown for each cell type (grey or single colored tracks) along with a representative domainogram immediately below. Subtraction tracks, in $\log_2$ scale, are shown for each deletion clone interaction profile from Caco2 WT cells. Losses (above) and gains (below) in interactions from Caco2 WT are shown with respect to the y-axis.

Figure S10: Impact of the 185+2.7kb Δ5.1kb and 185+5.7kb Δ2.1kb deletions on interactions with the *CFTR* promoter in Caco2 cells. 4C-seq analysis of Caco2 WT (grey), 185+2.7kb Δ5.1kb clones (purple), or 185+5.7kb Δ2.1kb clones (gold) with the viewpoint at the *CFTR* promoter (red dotted line). Key *CFTR* CREs as well as the deletions are shown at the top and bottom. Read quantification tracks from an average of two replicates is shown for each cell type (grey or single colored tracks) along with a representative domainogram immediately below. Subtraction tracks, in $\log_2$ scale, are shown for each deletion clone interaction profile from Caco2 WT cells. Losses (above) and gains (below) in interactions from Caco2 WT are shown with respect to the y-axis.

Figure S11: Extended analysis of interactions of the 185+2.7kb Δ5.1kb deletion clones with the 5' TAD boundary in Caco2 cells. 4C-seq analysis of Caco2 WT (grey) and 185+2.7kb Δ5.1kb clones (purple) with the viewpoint at the -20.9kb insulator (red dotted line). Key *CFTR* CREs as well as the deletion are shown at the top. Read quantification tracks from an average of two replicates is shown for each cell type (grey and single colored tracks) along with a representative domainogram immediately below. Subtraction tracks, in $\log_2$ scale, are shown for each deletion clone interaction profile from Caco2 WT cells. Losses (above) and gains (below) in interactions from Caco2 WT are shown with respect to the y-axis. Regions of interest marked by horizontal bars or arrows.

Figure S12: Extended analysis of the interactions of the 185+5.7kb Δ2.1kb deletion clones with the -20.9kb insulator in Caco2 cells. 4C-seq analysis of Caco2 WT (grey) and 185+5.7kb Δ2.1kb clones (gold) with the viewpoint at the -20.9kb insulator (red dotted line). See Figure S11 legend for detailed description of tracks shown.

Figure S13: Extended analysis of the interactions of the 185+2.7kb Δ5.1kb deletion clones with *CFTR* intron 1 (185+19.5kb) in Caco2 cells. 4C-seq analysis of Caco2 WT (grey) and 185+2.7kb Δ5.1kb clones (purple) with the viewpoint at intron 1 (185+19.5kb) of *CFTR* (red dotted line). See Figure S11 legend for detailed description of tracks shown.

Figure S14: Extended analysis of the interactions of the 185+5.7kb Δ2.1kb deletion clones with *CFTR* intron 1 (185+19.5kb) in Caco2 cells. 4C-seq analysis of Caco2 WT (grey) and 185+5.7kb Δ2.1kb clones (gold) with the viewpoint at intron 1 (185+19.5kb) of *CFTR* (red dotted line). See Figure S11 legend for detailed description of tracks shown.

Figure S15: Extended analysis of the interactions of the 185+2.7kb Δ5.1kb deletion clones with the 3' TAD boundary in Caco2 cells. 4C-seq analysis of Caco2 WT (grey) and 185+2.7kb Δ5.1kb clones (purple) with the viewpoint at the 3' TAD boundary at +48.9kb (red dotted line). See Figure S11 legend for detailed description of tracks shown.

Figure S16: Extended analysis of the interactions of the 185+5.7kb Δ2.1kb deletion clones with the 3' TAD boundary in Caco2 cells. 4C-seq analysis of Caco2 WT (grey) and 185+5.7kb Δ2.1kb clones (gold) with the viewpoint at the 3' TAD boundary at +48.9kb (red dotted line). See Figure S11 legend for detailed description of tracks shown.

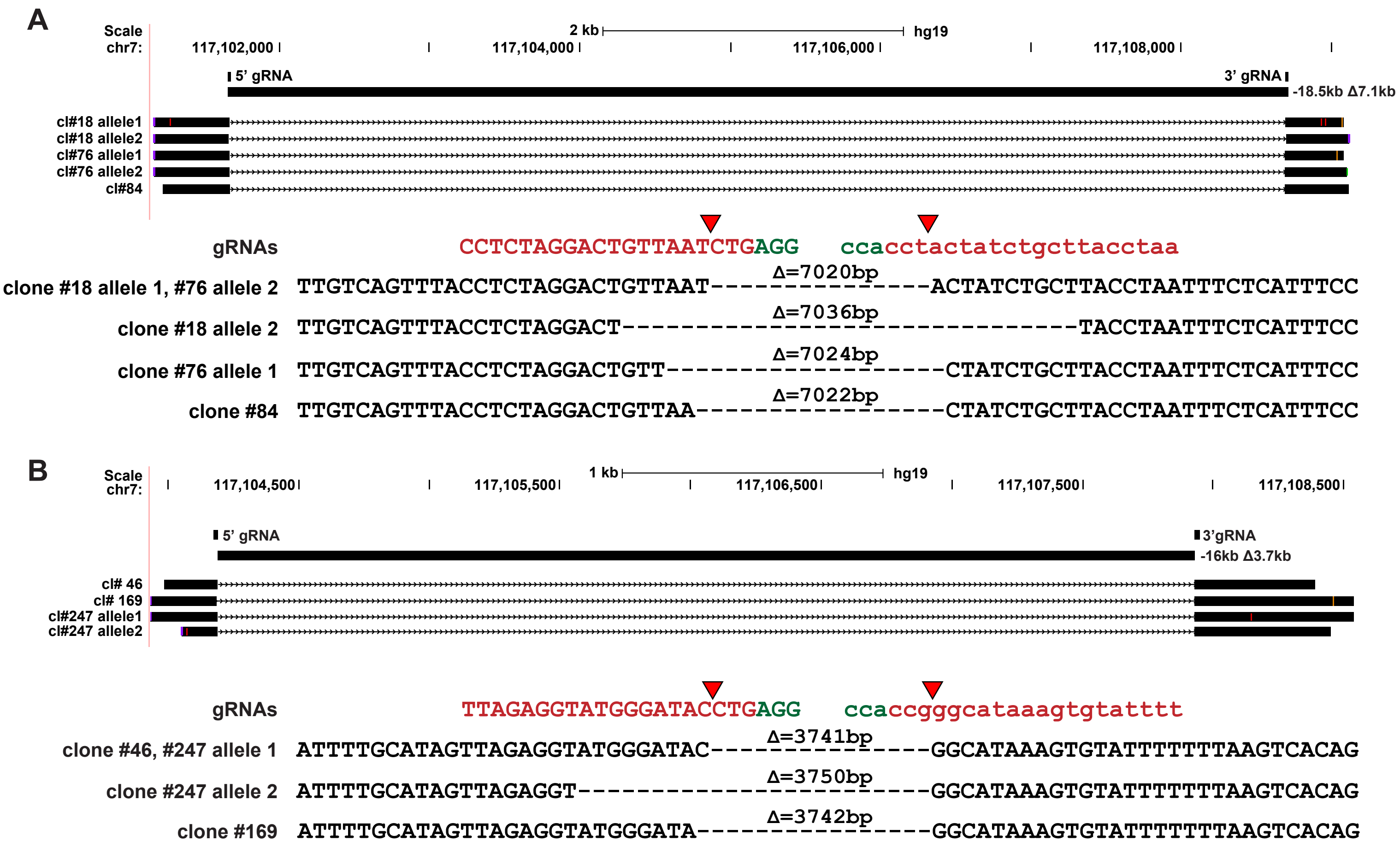
A
Scale
chr7:
117,102,000
2 kb
117,104,000
117,106,000
hg19
117,108,000
5' gRNA
3' gRNA
-18.5kb Δ7.1kb
cl#18 allele1
cl#18 allele2
cl#76 allele1
cl#76 allele2
cl#84
gRNAs
CCTCTAGGACTGTTAATCTGAGG
ccacctactatctgcttacctaa
clone #18 allele 1, #76 allele 2
TTGTCAGTTTACCTCTAGGACTGTTAAT----------------ACTATCTGCTTACCTAATTTCTCATTTCC
Δ=7020bp
clone #18 allele 2
TTGTCAGTTTACCTCTAGGACT--------------------------------TACCTAATTTCTCATTTCC
Δ=7036bp
clone #76 allele 1
TTGTCAGTTTACCTCTAGGACTGTT------------------CTATCTGCTTACCTAATTTCTCATTTCC
Δ=7024bp
clone #84
TTGTCAGTTTACCTCTAGGACTGTTAA----------------CTATCTGCTTACCTAATTTCTCATTTCC
Δ=7022bp
B
Scale
chr7:
117,104,500
117,105,500
1 kb
117,106,500
hg19
117,107,500
117,108,500
5' gRNA
3'gRNA
-16kb Δ3.7kb
cl# 46
cl# 169
cl#247 allele1
cl#247 allele2
gRNAs
TTAGAGGTATGGGATACCTGAGG
ccaccgggcataaagtgtatttt
clone #46, #247 allele 1
ATTTTGCATAGTTAGAGGTATGGGATAC----------------GGCATAAAGTGTATTTTTTTAAGTCACAG
Δ=3741bp
clone #247 allele 2
ATTTTGCATAGTTAGAGGT-------------------------GGCATAAAGTGTATTTTTTTAAGTCACAG
Δ=3750bp
clone #169
ATTTTGCATAGTTAGAGGTATGGGATA-----------------GGCATAAAGTGTATTTTTTTAAGTCACAG
Δ=3742bp

Figure S1

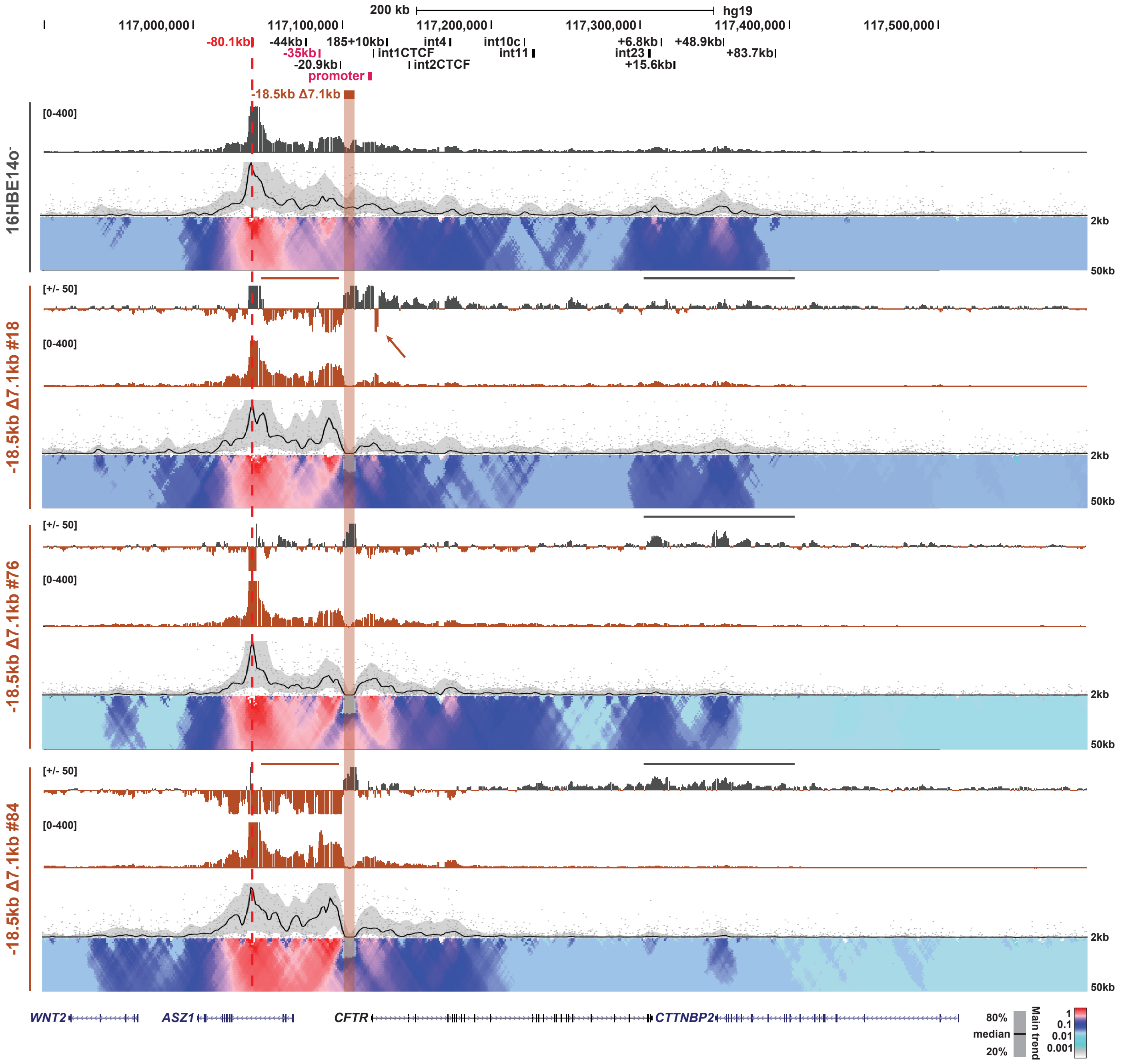
200 kb
hg19
117,000,000
117,100,000
117,200,000
117,300,000
117,400,000
117,500,000
-80.1kb
-44kb
-35kb
-20.9kb
185+10kb
int1CTCF
int2CTCF
promoter
int4
int10c
int11
+6.8kb
int23
+15.6kb
+48.9kb
+83.7kb
-18.5kb Δ7.1kb
16HBE14o-
[0-400]
2kb
50kb
-18.5kb Δ7.1kb #18
[+/- 50]
-18.5kb Δ7.1kb #76
-18.5kb Δ7.1kb #84
WNT2
ASZ1
CFTR
CTTNBP2
80%
median
20%
Main trend
1
0.1
0.01
0.001

Figure S2

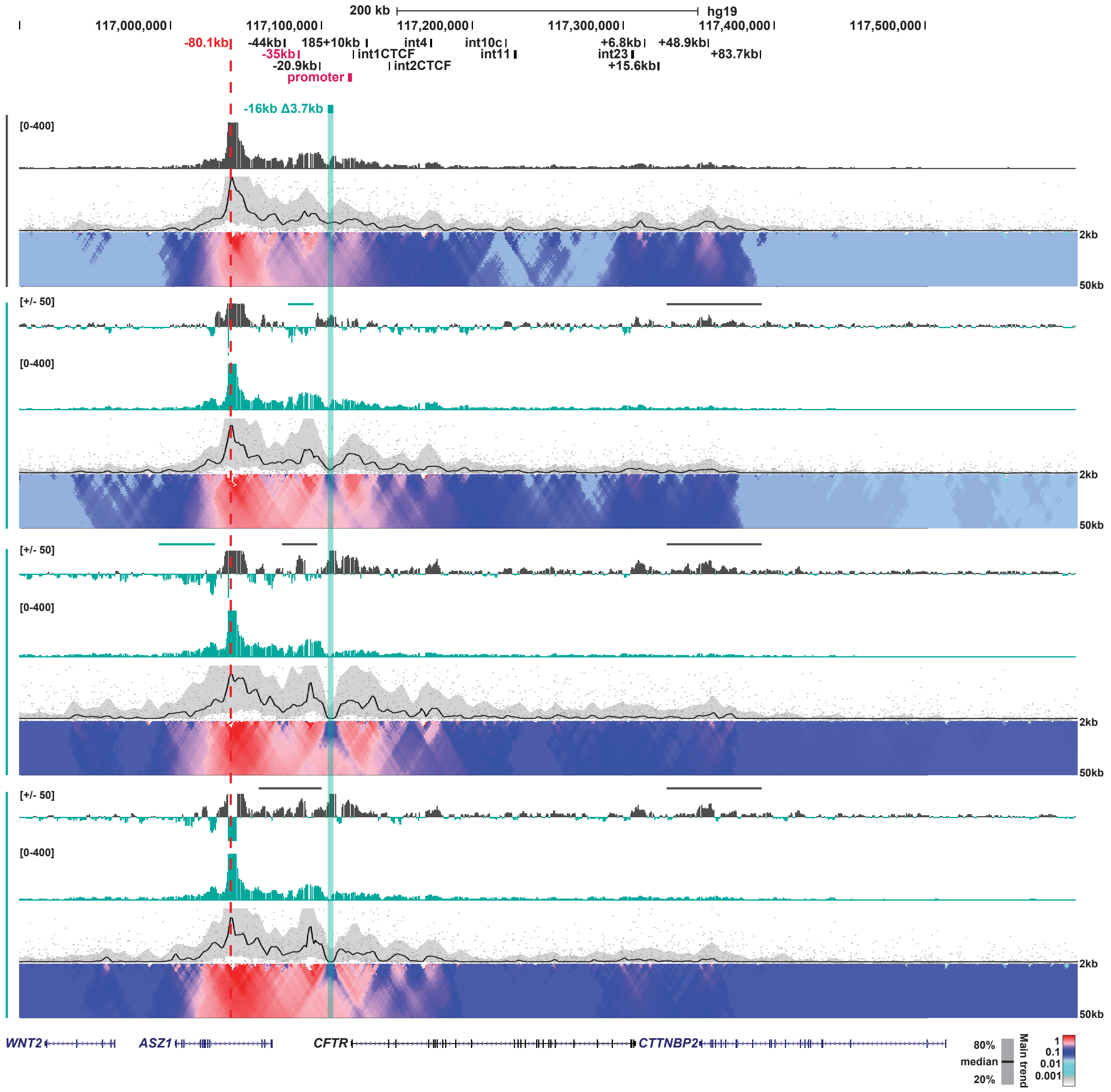

200 kb
hg19
117,000,000
117,100,000
117,200,000
117,300,000
117,400,000
117,500,000
-80.1kb
-44kb
185+10kb
int4
int10c
+6.8kb
+48.9kb
-35kb
int1CTCF
int11
int23
+83.7kb
-20.9kb
int2CTCF
+15.6kb
promoter
-16kb Δ3.7kb
16HBE14o⁻
[0-400]
2kb
50kb
-16kb Δ3.7kb #46
[+/- 50]
[0-400]
2kb
50kb
-16kb Δ3.7kb #169
[+/- 50]
[0-400]
2kb
50kb
-16kb Δ3.7kb #247
[+/- 50]
[0-400]
2kb
50kb
WNT2
ASZ1
CFTR
CTTNBP2
80%
median
20%
Main trend
1
0.1
0.01
0.001

Figure S3

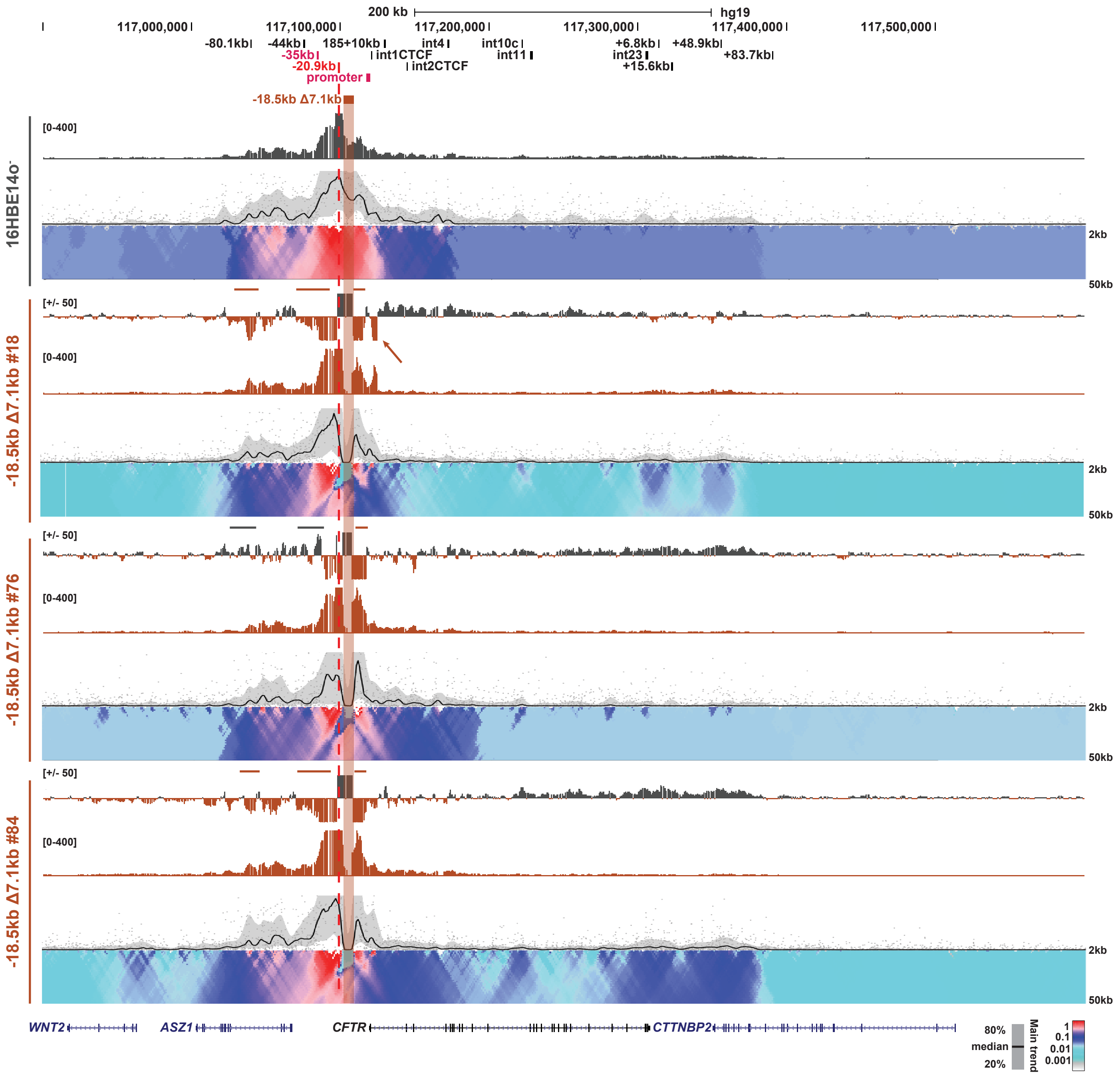
200 kb
hg19
117,000,000
117,100,000
117,200,000
117,300,000
117,400,000
117,500,000
-80.1kb
-44kb
185+10kb
int4
int10c
+6.8kb
+48.9kb
-35kb
int1CTCF
int11
int23
+83.7kb
-20.9kb
int2CTCF
+15.6kb
promoter
-18.5kb Δ7.1kb
16HBE14o-
[0-400]
2kb
50kb
-18.5kb Δ7.1kb #18
[+/- 50]
[0-400]
2kb
50kb
-18.5kb Δ7.1kb #76
[+/- 50]
[0-400]
2kb
50kb
-18.5kb Δ7.1kb #84
[+/- 50]
[0-400]
2kb
50kb
WNT2
ASZ1
CFTR
CTTNBP2
80%
median
20%
Main trend
1
0.1
0.01
0.001

Figure S4

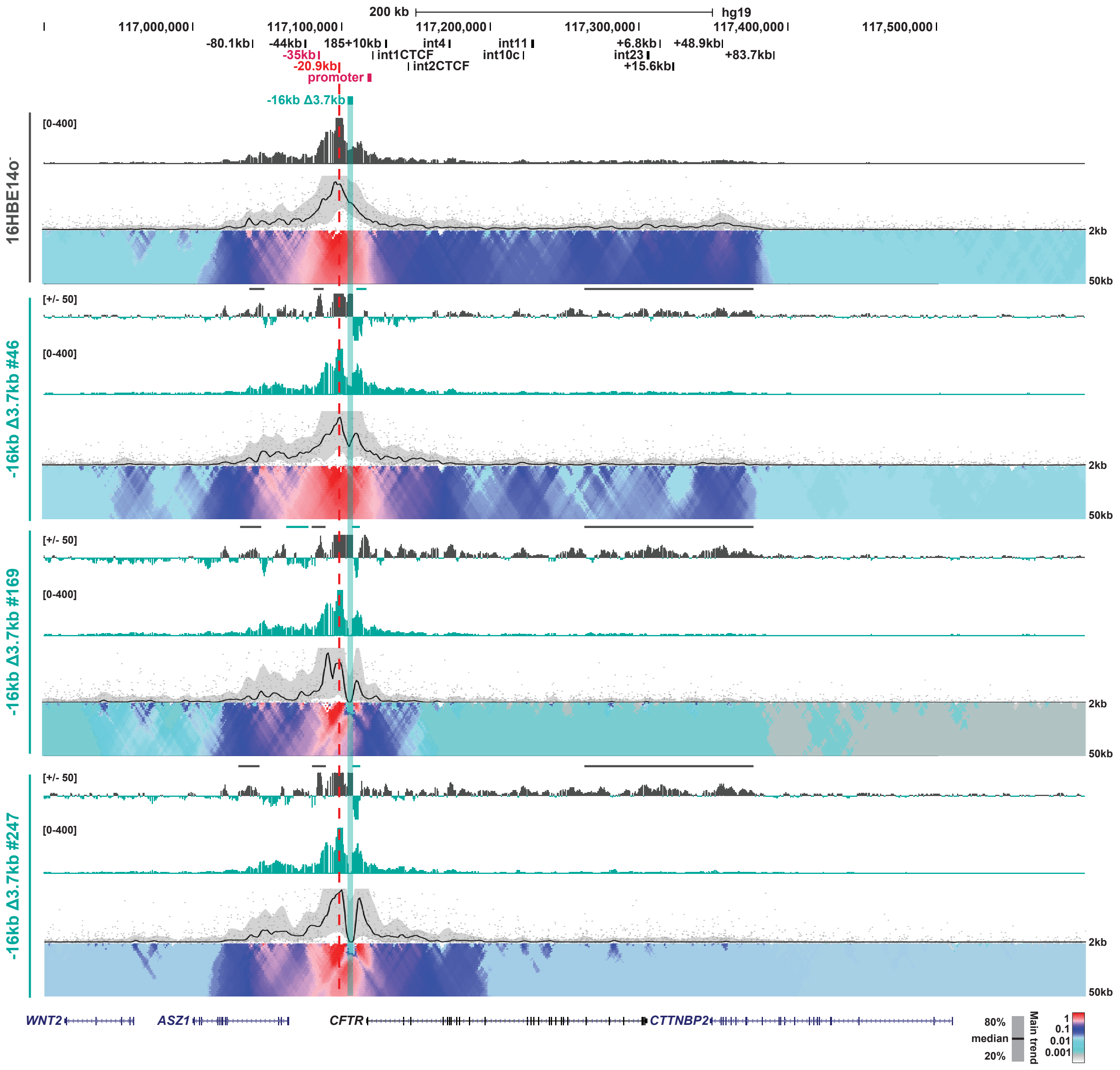
200 kb
hg19
117,000,000
117,100,000
117,200,000
117,300,000
117,400,000
117,500,000
-80.1kb
-44kb
-35kb
-20.9kb
promoter
185+10kb
int1CTCF
int2CTCF
int4
int11
int10c
+6.8kb
int23
+15.6kb
+48.9kb
+83.7kb
-16kb Δ3.7kb
16HBE14o-
[0-400]
[+/- 50]
2kb
50kb
-16kb Δ3.7kb #46
-16kb Δ3.7kb #169
-16kb Δ3.7kb #247
WNT2
ASZ1
CFTR
CTTNBP2
80%
median
20%
Main trend
1
0.1
0.01
0.001

Figure S5

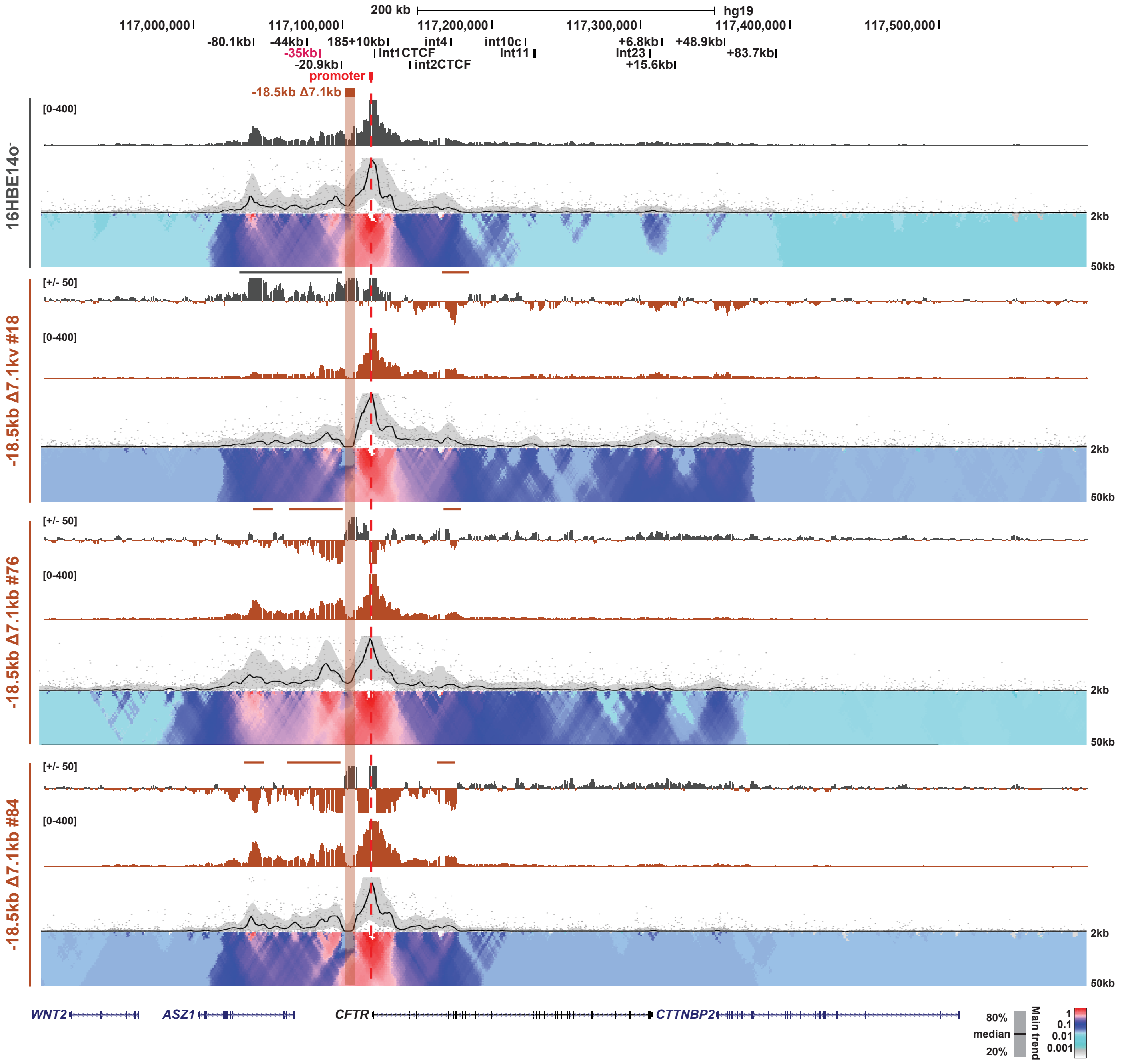
200 kb
hg19
117,000,000
117,100,000
117,200,000
117,300,000
117,400,000
117,500,000
-80.1kb
-44kb
185+10kb
int4
int10c
+6.8kb
+48.9kb
-35kb
int1CTCF
int11
int23
+83.7kb
-20.9kb
int2CTCF
+15.6kb
promoter
-18.5kb Δ7.1kb
16HBE14o-
[0-400]
2kb
50kb
[+/- 50]
-18.5kb Δ7.1kv #18
-18.5kb Δ7.1kb #76
-18.5kb Δ7.1kb #84
WNT2
ASZ1
CFTR
CTTNBP2
80%
median
20%
Main trend
1
0.1
0.01
0.001

Figure S6

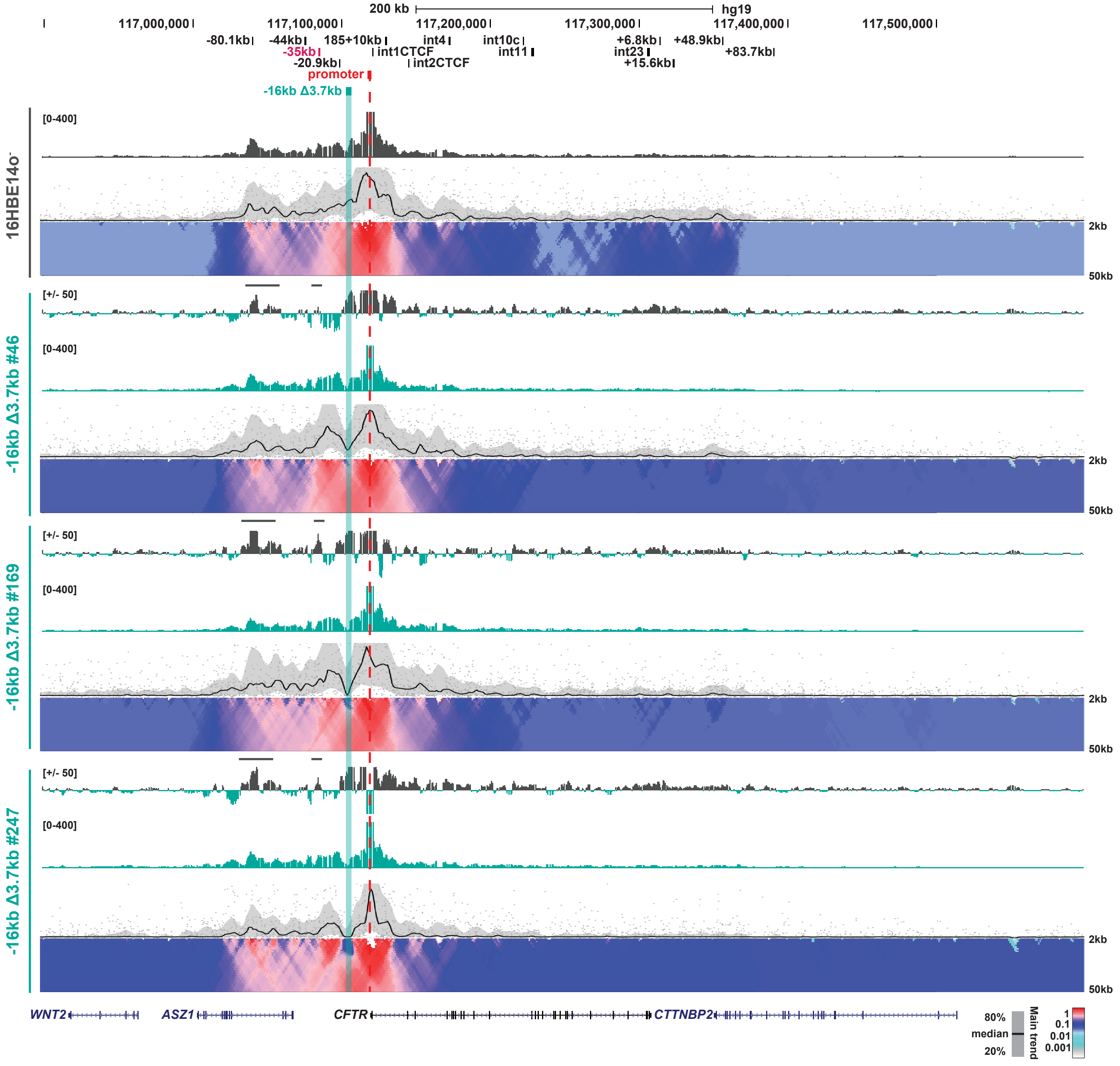

200 kb
hg19
117,000,000
117,100,000
117,200,000
117,300,000
117,400,000
117,500,000
-80.1kb
-44kb
-35kb
-20.9kb
185+10kb
int1CTCF
int2CTCF
int4
int10c
int11
+6.8kb
int23
+15.6kb
+48.9kb
+83.7kb
promoter
-16kb Δ3.7kb
16HBE14o-
-16kb Δ3.7kb #46
-16kb Δ3.7kb #169
-16kb Δ3.7kb #247
[0-400]
[+/- 50]
2kb
50kb
WNT2
ASZ1
CFTR
CTTNBP2
80%
median
20%
Main trend
1
0.1
0.01
0.001

Figure S7

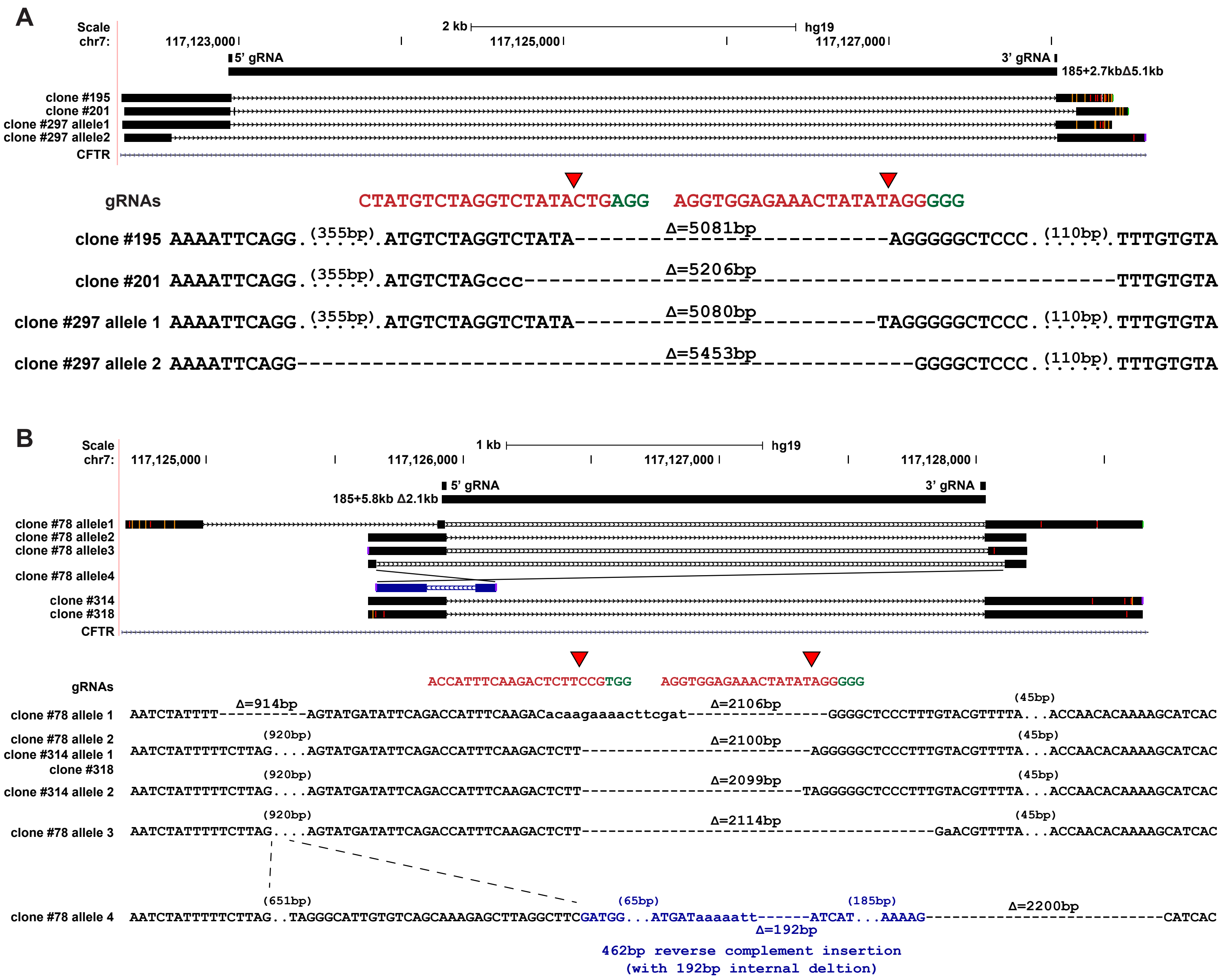
A
Scale
chr7:
2 kb
hg19
117,123,000
117,125,000
117,127,000
5' gRNA
3' gRNA
185+2.7kbΔ5.1kb
clone #195
clone #201
clone #297 allele1
clone #297 allele2
CFTR
gRNAs
CTATGTCTAGGTCTATACTGAGG
AGGTGGAGAAACTATATAGGGGG
clone #195 AAAATTCAGG.(355bp).ATGTCTAGGTCTATA----Δ=5081bp----AGGGGGCTCCC.(110bp).TTTGTGTA
clone #201 AAAATTCAGG.(355bp).ATGTCTAGccc----Δ=5206bp----TTTGTGTA
clone #297 allele 1 AAAATTCAGG.(355bp).ATGTCTAGGTCTATA----Δ=5080bp----TAGGGGGCTCCC.(110bp).TTTGTGTA
clone #297 allele 2 AAAATTCAGG----Δ=5453bp----GGGGCTCCC.(110bp).TTTGTGTA
B
Scale
chr7:
1 kb
hg19
117,125,000
117,126,000
117,127,000
117,128,000
5' gRNA
3' gRNA
185+5.8kb Δ2.1kb
clone #78 allele1
clone #78 allele2
clone #78 allele3
clone #78 allele4
clone #314
clone #318
CFTR
gRNAs
ACCATTTCAAGACTCTTCCGTGG
AGGTGGAGAAACTATATAGGGGG
clone #78 allele 1 AATCTATTTT---Δ=914bp---AGTATGATATTCAGACCATTTCAAGACacaagaaaacttcgat----Δ=2106bp----GGGGCTCCCTTTGTACGTTTTA...(45bp)...ACCAACACAAAAGCATCAC
clone #78 allele 2 / clone #314 allele 1 / clone #318 AATCTATTTTTCTTAG....(920bp)....AGTATGATATTCAGACCATTTCAAGACTCTT----Δ=2100bp----AGGGGGCTCCCTTTGTACGTTTTA...(45bp)...ACCAACACAAAAGCATCAC
clone #314 allele 2 AATCTATTTTTCTTAG....(920bp)....AGTATGATATTCAGACCATTTCAAGACTCTT----Δ=2099bp----TAGGGGGCTCCCTTTGTACGTTTTA...(45bp)...ACCAACACAAAAGCATCAC
clone #78 allele 3 AATCTATTTTTCTTAG....(920bp)....AGTATGATATTCAGACCATTTCAAGACTCTT----Δ=2114bp----GaACGTTTTA...(45bp)...ACCAACACAAAAGCATCAC
clone #78 allele 4 AATCTATTTTTCTTAG..(651bp)..TAGGGCATTGTGTCAGCAAAGAGCTTAGGCTTCGATGG...(65bp)...ATGATaaaaatt------ATCAT...(185bp)...AAAAG----Δ=2200bp----CATCAC
Δ=192bp
462bp reverse complement insertion
(with 192bp internal deltion)

Figure S8

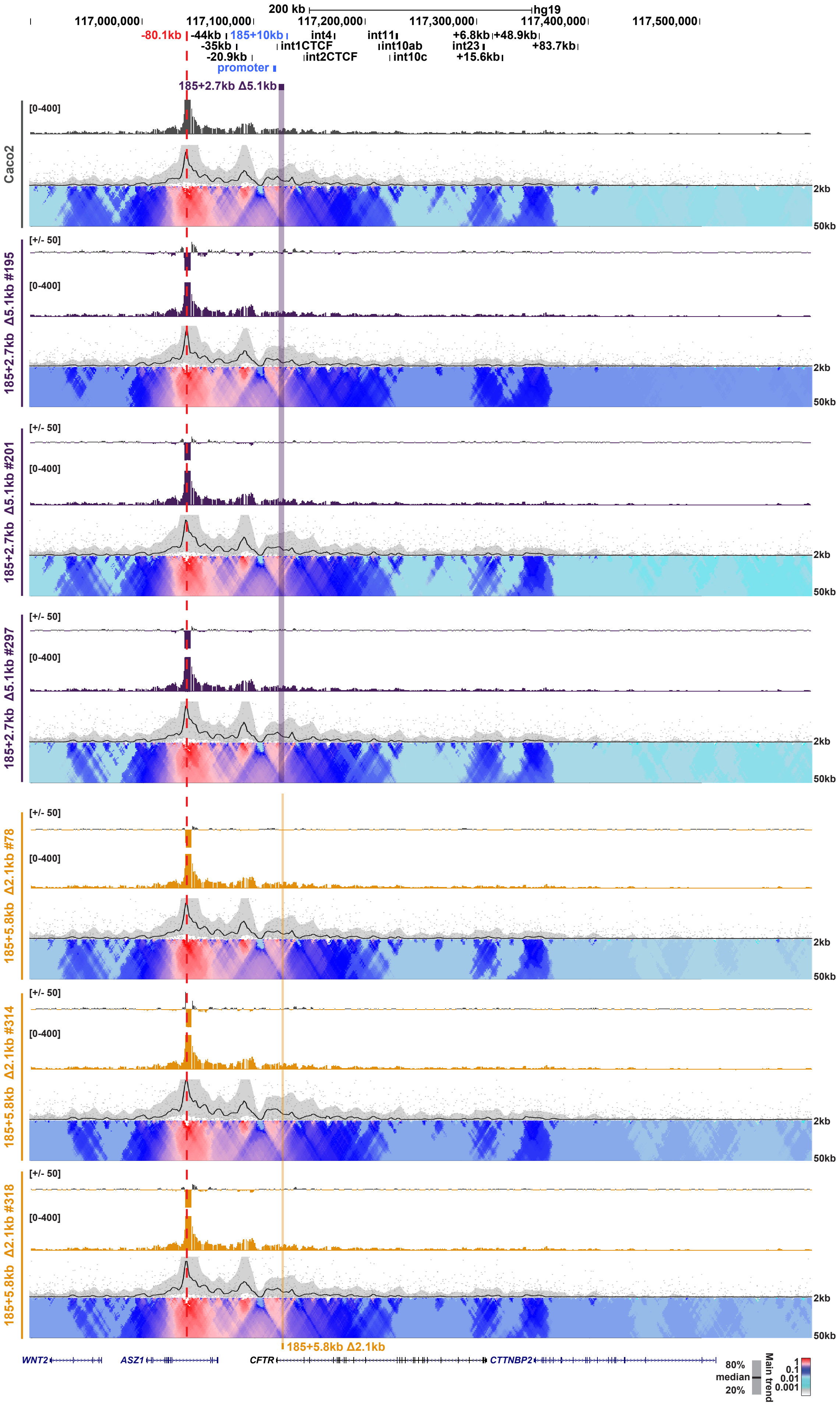

200 kb
hg19
117,000,000
117,100,000
117,200,000
117,300,000
117,400,000
117,500,000
-80.1kb
-44kb
185+10kb
int4
int11
+6.8kb
+48.9kb
-35kb
int1CTCF
int10ab
int23
+83.7kb
-20.9kb
int2CTCF
int10c
+15.6kb
promoter
185+2.7kb Δ5.1kb
Caco2
[0-400]
2kb
50kb
185+2.7kb Δ5.1kb #195
[+/- 50]
185+2.7kb Δ5.1kb #201
185+2.7kb Δ5.1kb #297
185+5.8kb Δ2.1kb #78
185+5.8kb Δ2.1kb #314
185+5.8kb Δ2.1kb #318
185+5.8kb Δ2.1kb
WNT2
ASZ1
CFTR
CTTNBP2
80%
median
20%
Main trend
1
0.1
0.01
0.001

Figure S9

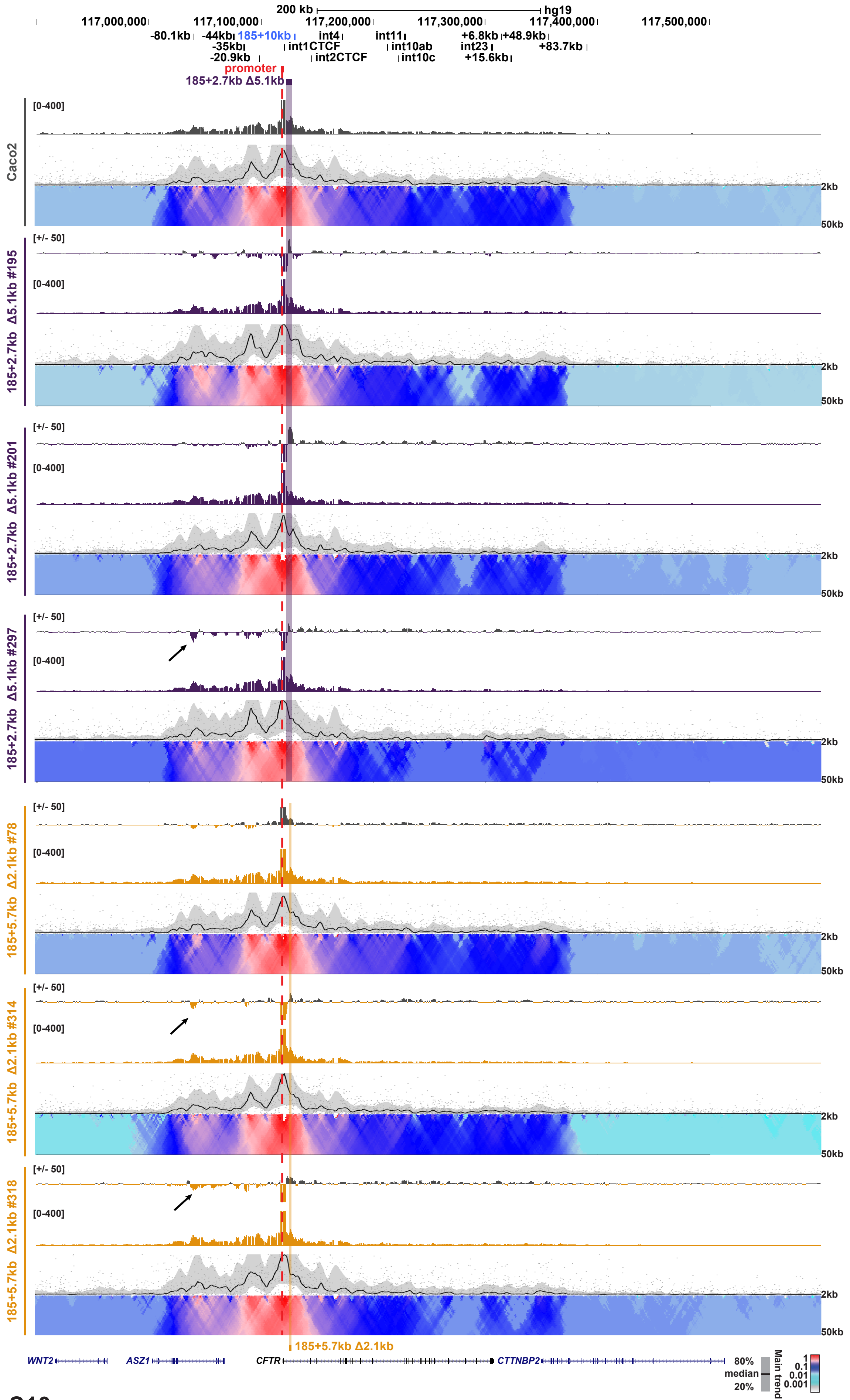

200 kb
hg19
117,000,000
117,100,000
117,200,000
117,300,000
117,400,000
117,500,000
-80.1kb
-44kb
185+10kb
int4
int11
+6.8kb
+48.9kb
-35kb
int1CTCF
int10ab
int23
+83.7kb
-20.9kb
int2CTCF
int10c
+15.6kb
promoter
185+2.7kb Δ5.1kb
Caco2
[0-400]
[+/- 50]
185+2.7kb Δ5.1kb #195
185+2.7kb Δ5.1kb #201
185+2.7kb Δ5.1kb #297
185+5.7kb Δ2.1kb #78
185+5.7kb Δ2.1kb #314
185+5.7kb Δ2.1kb #318
2kb
50kb
185+5.7kb Δ2.1kb
WNT2
ASZ1
CFTR
CTTNBP2
80%
median
20%
Main trend
1
0.1
0.01
0.001

Figure S10

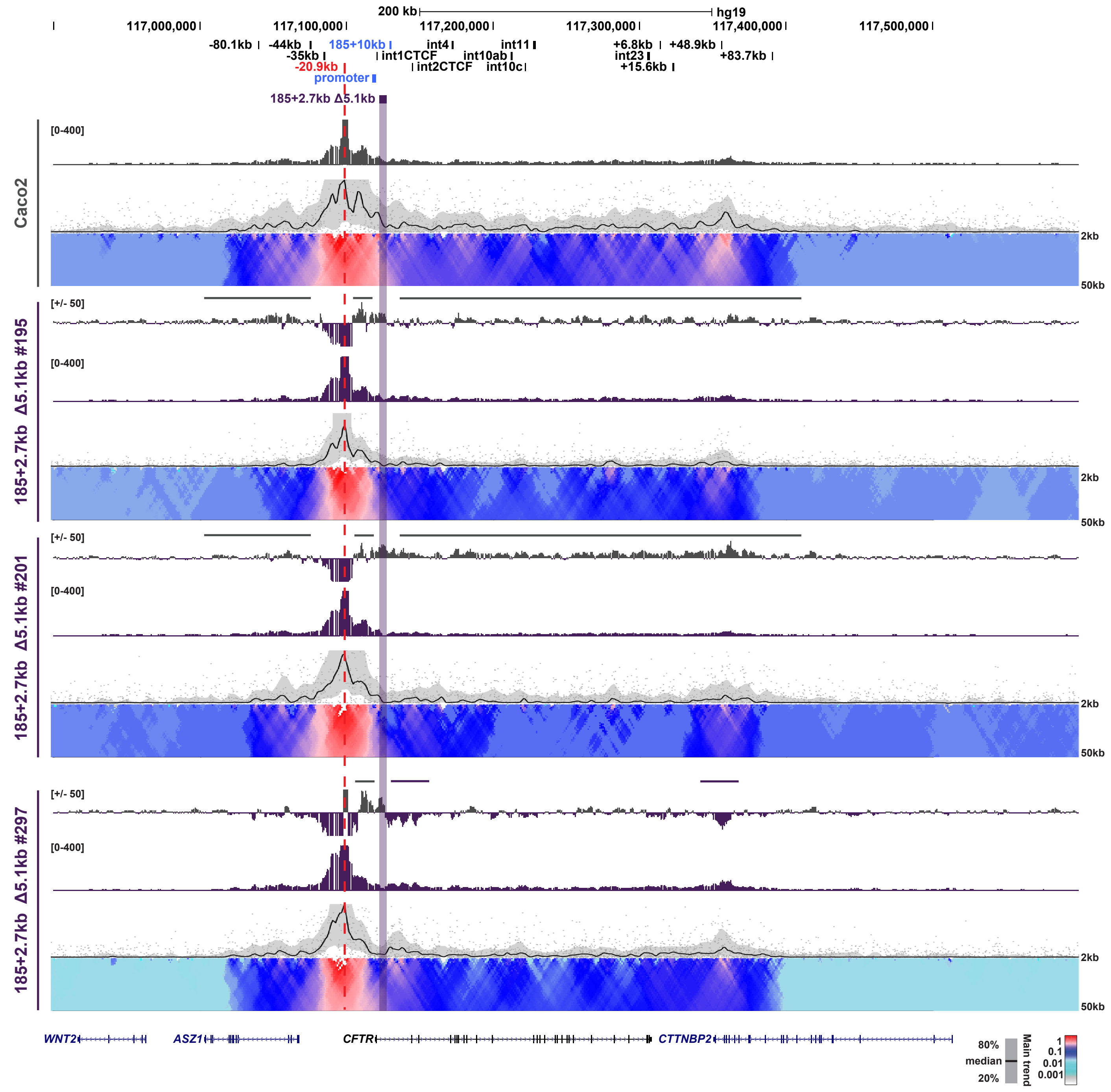

200 kb
hg19
117,000,000
117,100,000
117,200,000
117,300,000
117,400,000
117,500,000
-80.1kb
-44kb
-35kb
-20.9kb
185+10kb
promoter
int1CTCF
int2CTCF
int4
int10ab
int10c
int11
+6.8kb
int23
+15.6kb
+48.9kb
+83.7kb
185+2.7kb Δ5.1kb
Caco2
[0-400]
2kb
50kb
185+2.7kb Δ5.1kb #195
[+/- 50]
185+2.7kb Δ5.1kb #201
185+2.7kb Δ5.1kb #297
WNT2
ASZ1
CFTR
CTTNBP2
80%
median
20%
Main trend
1
0.1
0.01
0.001

Figure S11

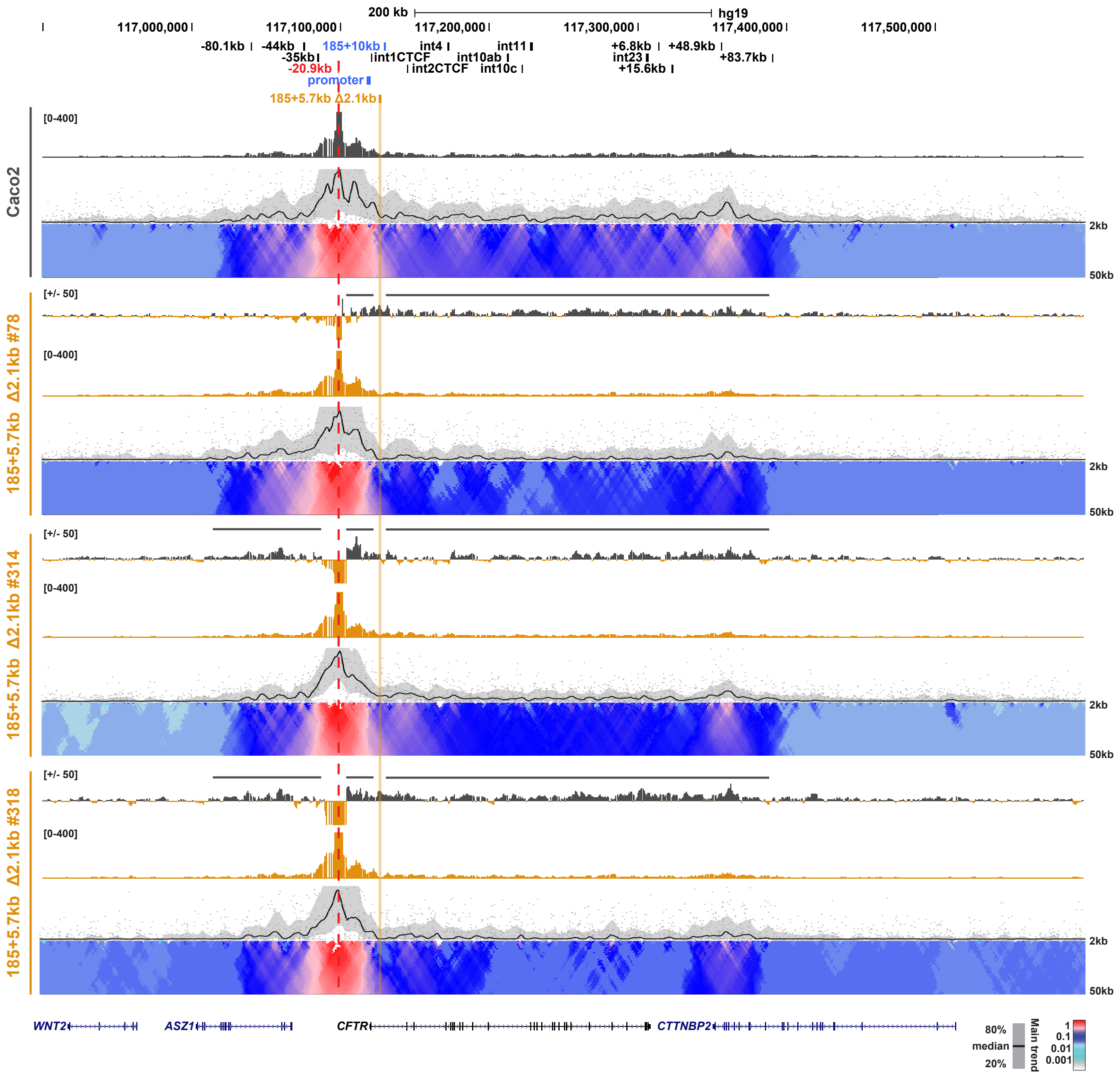
200 kb
hg19
117,000,000
117,100,000
117,200,000
117,300,000
117,400,000
117,500,000
-80.1kb
-44kb
-35kb
-20.9kb
185+10kb
promoter
int1CTCF
int2CTCF
int4
int10ab
int10c
int11
+6.8kb
int23
+15.6kb
+48.9kb
+83.7kb
185+5.7kb Δ2.1kb
Caco2
[0-400]
2kb
50kb
185+5.7kb Δ2.1kb #78
[+/- 50]
[0-400]
2kb
50kb
185+5.7kb Δ2.1kb #314
[+/- 50]
[0-400]
2kb
50kb
185+5.7kb Δ2.1kb #318
[+/- 50]
[0-400]
2kb
50kb
WNT2
ASZ1
CFTR
CTTNBP2
80%
median
20%
Main trend
1
0.1
0.01
0.001

Figure S12

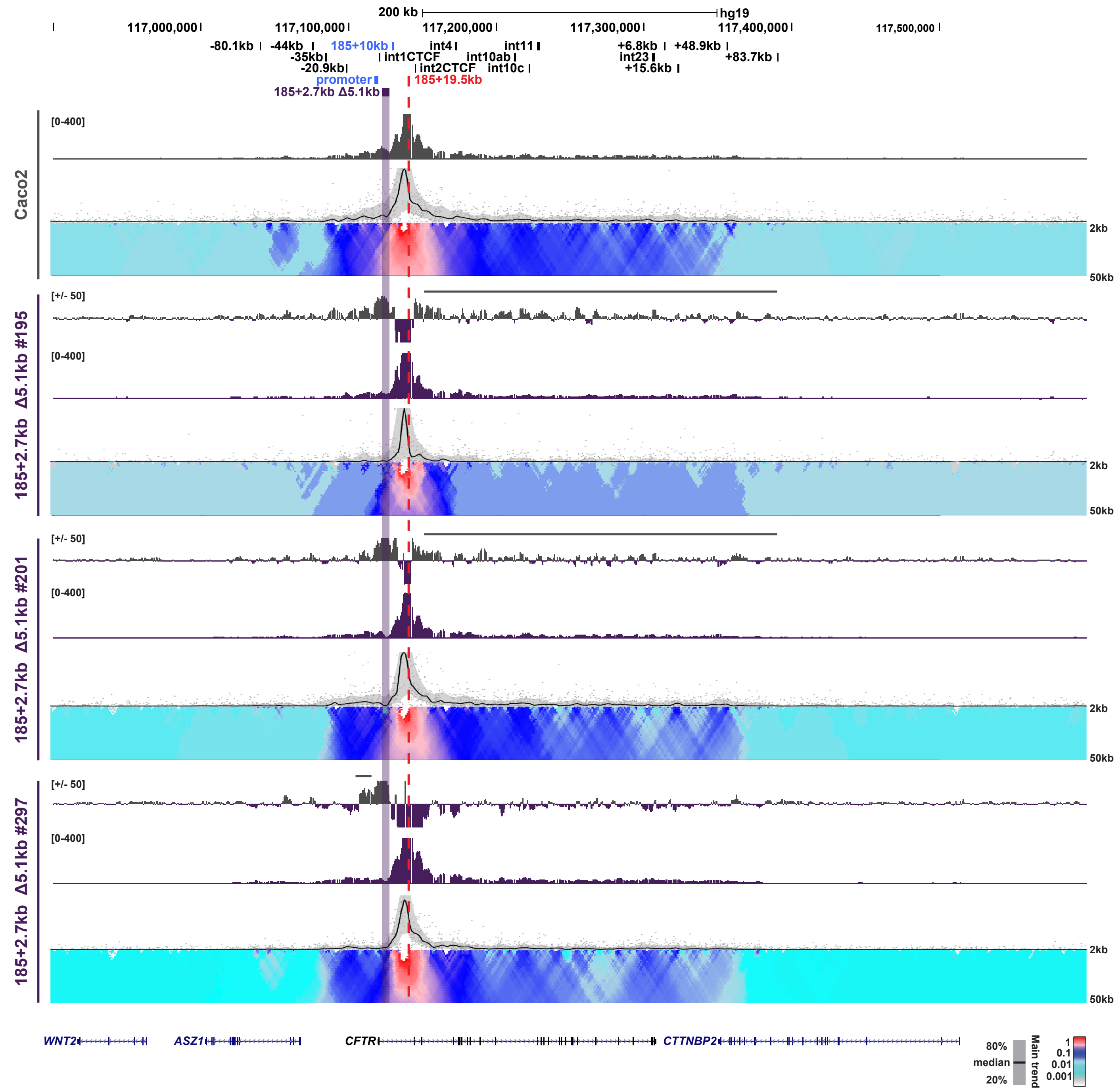

200 kb
hg19
117,000,000
117,100,000
117,200,000
117,300,000
117,400,000
117,500,000
-80.1kb
-44kb
-35kb
-20.9kb
185+10kb
int1CTCF
int2CTCF
int4
int10ab
int10c
int11
+6.8kb
int23
+15.6kb
+48.9kb
+83.7kb
promoter
185+19.5kb
185+2.7kb Δ5.1kb
Caco2
185+2.7kb Δ5.1kb #195
185+2.7kb Δ5.1kb #201
185+2.7kb Δ5.1kb #297
[0-400]
[+/- 50]
2kb
50kb
WNT2
ASZ1
CFTR
CTTNBP2
80%
median
20%
Main trend
1
0.1
0.01
0.001

Figure S13

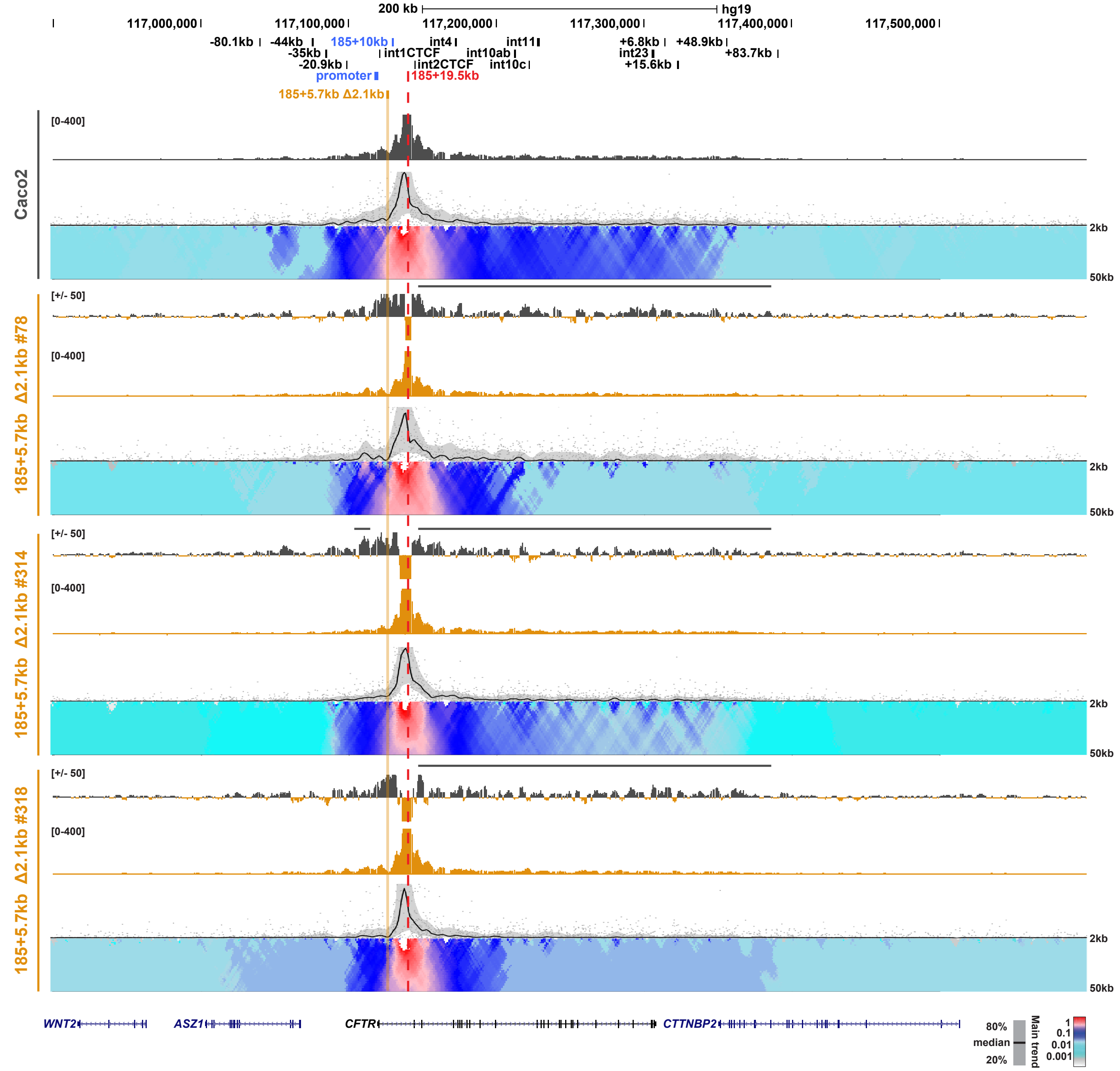
200 kb
hg19
117,000,000
117,100,000
117,200,000
117,300,000
117,400,000
117,500,000
-80.1kb
-44kb
-35kb
-20.9kb
185+10kb
promoter
int1CTCF
int2CTCF
185+19.5kb
int4
int11
int10ab
int10c
+6.8kb
+48.9kb
int23
+15.6kb
+83.7kb
185+5.7kb Δ2.1kb
Caco2
[0-400]
2kb
50kb
185+5.7kb Δ2.1kb #78
[+/- 50]
[0-400]
2kb
50kb
185+5.7kb Δ2.1kb #314
[+/- 50]
[0-400]
2kb
50kb
185+5.7kb Δ2.1kb #318
[+/- 50]
[0-400]
2kb
50kb
WNT2
ASZ1
CFTR
CTTNBP2
80%
median
20%
Main trend
1
0.1
0.01
0.001

Figure S14

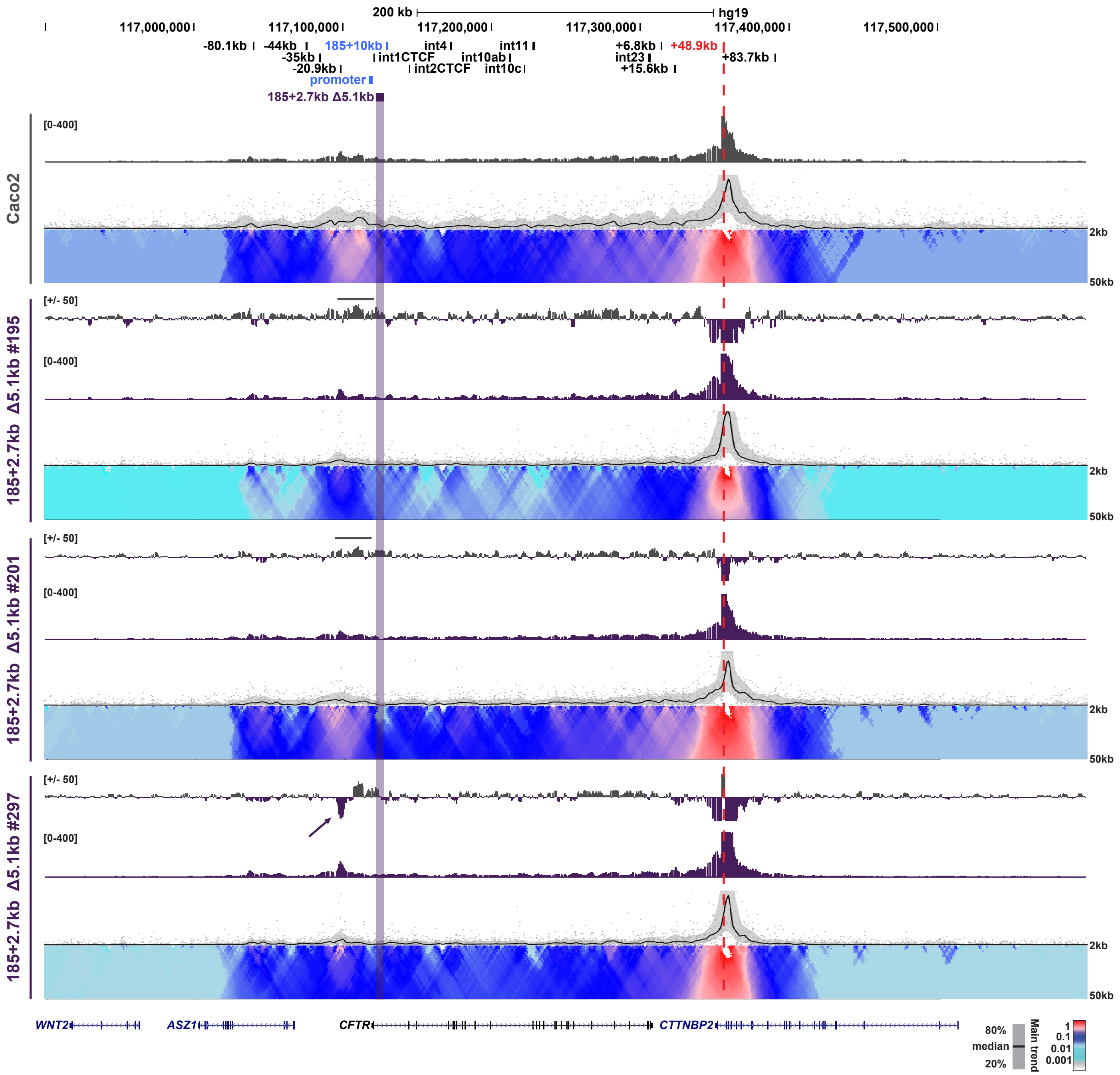
200 kb
hg19
117,000,000
117,100,000
117,200,000
117,300,000
117,400,000
117,500,000
-80.1kb
-44kb
-35kb
-20.9kb
185+10kb
promoter
int1CTCF
int2CTCF
int4
int10ab
int10c
int11
+6.8kb
int23
+15.6kb
+48.9kb
+83.7kb
185+2.7kb Δ5.1kb
Caco2
[0-400]
2kb
50kb
185+2.7kb Δ5.1kb #195
[+/- 50]
[0-400]
185+2.7kb Δ5.1kb #201
185+2.7kb Δ5.1kb #297
WNT2
ASZ1
CFTR
CTTNBP2
80%
median
20%
Main trend
1
0.1
0.01
0.001

Figure S15

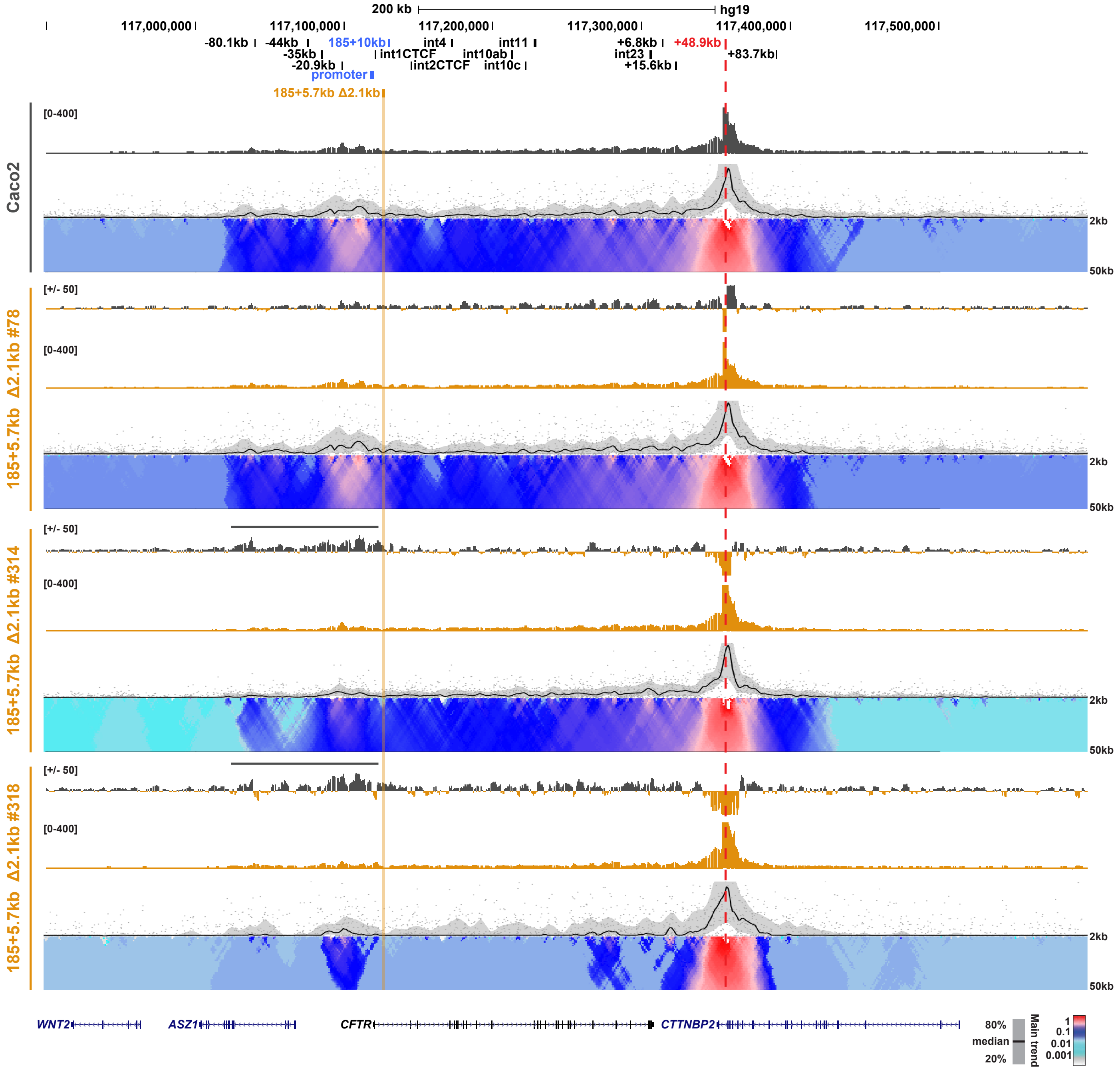
200 kb
hg19
117,000,000
117,100,000
117,200,000
117,300,000
117,400,000
117,500,000
-80.1kb
-44kb
-35kb
-20.9kb
185+10kb
promoter
int1CTCF
int2CTCF
int4
int10ab
int10c
int11
int23
+6.8kb
+15.6kb
+48.9kb
+83.7kb
185+5.7kb Δ2.1kb
Caco2
[0-400]
2kb
50kb
185+5.7kb Δ2.1kb #78
[+/- 50]
[0-400]
185+5.7kb Δ2.1kb #314
185+5.7kb Δ2.1kb #318
WNT2
ASZ1
CFTR
CTTNBP2
80%
median
20%
Main trend
1
0.1
0.01
0.001

Figure S16

Table S1: Oligonucleotides

16HBE14o- -18.5kb Δ7.1kb

Name	Sequence (5'>3')
CFTR -18.5kb gBlock	TGTACAAAAAAGCAGGCTTTAAAGGAACCAATTCAGTCGACTGGATCCGGTACCAAGGTCGGGCAGGAAGAGGGCCTATTTCCCATGATTCCTTCATATTTGCATAT<br>ACGATACAAGGCTGTTAGAGAGATAATTAGAATTAATTTGACTGTAAACACAAAGATATTAGTACAAAATACGTGACGTAGAAAGTAATAATTTCTTGGGTAGTTTG<br>CAGTTTTAAAATTATGTTTTAAAATGGACTATCATATGCTTACCGTAACTTGAAAGTATTTCGATTTCTTGGCTTTATATATCTTGTGGAAAGGACGAAACACCG**CC**<br>**TCTAGGACTGTTAATCTG**GTTTTAGAGCTAGAAATAGCAAGTTAAAATAAGGCTAGTCCGTTATCAACTTGAAAAAGTGGCACCGAGTCGGTGCTTTTTTTCTAGAC<br>CCAGCTTTCTTGTACAAAGTTGGCATTA
CFTR -11.4kb gBlock	TGTACAAAAAAGCAGGCTTTAAAGGAACCAATTCAGTCGACTGGATCCGGTACCAAGGTCGGGCAGGAAGAGGGCCTATTTCCCATGATTCCTTCATATTTGCATAT<br>ACGATACAAGGCTGTTAGAGAGATAATTAGAATTAATTTGACTGTAAACACAAAGATATTAGTACAAAATACGTGACGTAGAAAGTAATAATTTCTTGGGTAGTTTG<br>CAGTTTTAAAATTATGTTTTAAAATGGACTATCATATGCTTACCGTAACTTGAAAGTATTTCGATTTCTTGGCTTTATATATCTTGTGGAAAGGACGAAACACCG**TT**<br>**AGGTAAGCAGATAGTAGG**GTTTTAGAGCTAGAAATAGCAAGTTAAAATAAGGCTAGTCCGTTATCAACTTGAAAAAGTGGCACCGAGTCGGTGCTTTTTTTCTAGAC<br>CCAGCTTTCTTGTACAAAGTTGGCATTA
del7.1kb F1	TGGATGATCTTTGGTGCCCT
del7.1kb R1	GAGCAATCGTGAATGGGAAT
del7.1kb F2	ATGGTCCTGGGTTGGCATAA
del7.1kb R2	AGGGGAAGGGTTGAATAGGC

16HBE14o- -16kb Δ3.7kb

Name	Sequence (5'>3')
CFTR -16kb gBlock	TGTACAAAAAAGCAGGCTTTAAAGGAACCAATTCAGTCGACTGGATCCGGTACCAAGGTCGGGCAGGAAGAGGGCCTATTTCCCATGATTCCTTCATATTTGCATAT<br>ACGATACAAGGCTGTTAGAGAGATAATTAGAATTAATTTGACTGTAAACACAAAGATATTAGTACAAAATACGTGACGTAGAAAGTAATAATTTCTTGGGTAGTTTG<br>CAGTTTTAAAATTATGTTTTAAAATGGACTATCATATGCTTACCGTAACTTGAAAGTATTTCGATTTCTTGGCTTTATATATCTTGTGGAAAGGACGAAACACCG**TT**<br>**AGAGGTATGGGATACCTG**GTTTTAGAGCTAGAAATAGCAAGTTAAAATAAGGCTAGTCCGTTATCAACTTGAAAAAGTGGCACCGAGTCGGTGCTTTTTTTCTAGAC<br>CCAGCTTTCTTGTACAAAGTTGGCATTA
CFTR -12.2kb gBlock	TGTACAAAAAAGCAGGCTTTAAAGGAACCAATTCAGTCGACTGGATCCGGTACCAAGGTCGGGCAGGAAGAGGGCCTATTTCCCATGATTCCTTCATATTTGCATAT<br>ACGATACAAGGCTGTTAGAGAGATAATTAGAATTAATTTGACTGTAAACACAAAGATATTAGTACAAAATACGTGACGTAGAAAGTAATAATTTCTTGGGTAGTTTG<br>CAGTTTTAAAATTATGTTTTAAAATGGACTATCATATGCTTACCGTAACTTGAAAGTATTTCGATTTCTTGGCTTTATATATCTTGTGGAAAGGACGAAACACCG**AA**<br>**ATACACTTTATGCCCGG**GTTTTAGAGCTAGAAATAGCAAGTTAAAATAAGGCTAGTCCGTTATCAACTTGAAAAAGTGGCACCGAGTCGGTGCTTTTTTTCTAGACC<br>CAGCTTTCTTGTACAAAGTTGGCATTA
del2.1kb F1	CTGCACTATGAGGCACATGAA
del2.1kb F1.1	ACCCATGGAACAGGACAGAG
del2.1kb R1	ATGCTTTCGTGCGCTTTAGT
del2.1kb F2	ATGGTCCTGGGTTGGCATAA
del2.1kb R2	AGGGGAAGGGTTGAATAGGC

Caco2 c.185+2.7kb Δ5.1kb

Name	Sequence (5'>3')
CFTR c.185+2.7kb gBlock	TGTACAAAAAAGCAGGCTTTAAAGGAACCAATTCAGTCGACTGGATCCGGTACCAAGGTCGGGCAGGAAGAGGGCCTATTTCCCATGATTCCTTCATATTTGCATAT<br>ACGATACAAGGCTGTTAGAGAGATAATTAGAATTAATTTGACTGTAAACACAAAGATATTAGTACAAAATACGTGACGTAGAAAGTAATAATTTCTTGGGTAGTTTG<br>CAGTTTTAAAATTATGTTTTAAAATGGACTATCATATGCTTACCGTAACTTGAAAGTATTTCGATTTCTTGGCTTTATATATCTTGTGGAAAGGACGAAACACCG**TA**<br>**TGTCTAGGTCTATACTG**GTTTTAGAGCTAGAAATAGCAAGTTAAAATAAGGCTAGTCCGTTATCAACTTGAAAAAGTGGCACCGAGTCGGTGCTTTTTTTCTAGACC<br>CAGCTTTCTTGTACAAAGTTGGCATTA
CFTR c.185+7.8kb gBlock	TGTACAAAAAAGCAGGCTTTAAAGGAACCAATTCAGTCGACTGGATCCGGTACCAAGGTCGGGCAGGAAGAGGGCCTATTTCCCATGATTCCTTCATATTTGCATAT<br>ACGATACAAGGCTGTTAGAGAGATAATTAGAATTAATTTGACTGTAAACACAAAGATATTAGTACAAAATACGTGACGTAGAAAGTAATAATTTCTTGGGTAGTTTG<br>CAGTTTTAAAATTATGTTTTAAAATGGACTATCATATGCTTACCGTAACTTGAAAGTATTTCGATTTCTTGGCTTTATATATCTTGTGGAAAGGACGAAACACCG**GG**<br>**TGGAGAAACTATATAGG**GTTTTAGAGCTAGAAATAGCAAGTTAAAATAAGGCTAGTCCGTTATCAACTTGAAAAAGTGGCACCGAGTCGGTGCTTTTTTTCTAGACC<br>CAGCTTTCTTGTACAAAGTTGGCATTA
del5.1kb F1	TGTTTCACATGGCCTTACCA
del5.1kb R1	AGTCACTTGGAAGCAAGTCCA
del5.1kb F2	GAATCCCAGGGGTACTGACA
del5.1kb R2	TGGGAAAATGCAAGACACAA

Caco2 c.185+5.7kb Δ2.1kb

Name	Sequence (5'>3')
CFTR c.185+5.7kb gBlock	TGTACAAAAAAGCAGGCTTTAAAGGAACCAATTCAGTCGACTGGATCCGGTACCAAGGTCGGGCAGGAAGAGGGCCTATTTCCCATGATTCCTTCATATTTGCATAT<br>ACGATACAAGGCTGTTAGAGAGATAATTAGAATTAATTTGACTGTAAACACAAAGATATTAGTACAAAATACGTGACGTAGAAAGTAATAATTTCTTGGGTAGTTTG<br>CAGTTTTAAAATTATGTTTTAAAATGGACTATCATATGCTTACCGTAACTTGAAAGTATTTCGATTTCTTGGCTTTATATATCTTGTGGAAAGGACGAAACACC**GCC**<br>**ATTTCAAGACTCTTCCG**GTTTTAGAGCTAGAAATAGCAAGTTAAAATAAGGCTAGTCCGTTATCAACTTGAAAAAGTGGCACCGAGTCGGTGCTTTTTTTCTAGACC<br>CAGCTTTCTTGTACAAAGTTGGCATTA
CFTR c.185+7.8kb gBlock	TGTACAAAAAAGCAGGCTTTAAAGGAACCAATTCAGTCGACTGGATCCGGTACCAAGGTCGGGCAGGAAGAGGGCCTATTTCCCATGATTCCTTCATATTTGCATAT<br>ACGATACAAGGCTGTTAGAGAGATAATTAGAATTAATTTGACTGTAAACACAAAGATATTAGTACAAAATACGTGACGTAGAAAGTAATAATTTCTTGGGTAGTTTG<br>CAGTTTTAAAATTATGTTTTAAAATGGACTATCATATGCTTACCGTAACTTGAAAGTATTTCGATTTCTTGGCTTTATATATCTTGTGGAAAGGACGAAACACCG**GG**<br>**TGGAGAAACTATATAGG**GTTTTAGAGCTAGAAATAGCAAGTTAAAATAAGGCTAGTCCGTTATCAACTTGAAAAAGTGGCACCGAGTCGGTGCTTTTTTTCTAGACC<br>CAGCTTTCTTGTACAAAGTTGGCATTA
CFTR c.185+5.7kb gRNA (for RNP)	ACCATTTCAAGACTCTTCCG
CFTR c.185+7.8kb gRNA (for RNP)	AGGTGGAGAAACTATATAGG
del2.1kb F1	TAGGGCATTGTGTCAGCAAA
del2.1kb R1	CAATGAAGCCCTCGAGAAAG
del2.1kb F2	GAATCCCAGGGGTACTGACA
del2.1kb R2	TGGCCCACAAAGCTGAAAATATT
del2.1kb R2.1	TGGGAAAATGCAAGACACAA

TaqMan Assay

Gene	Sequence (5'>3')
CFTR	AGCTGTCAAGCCGTGTTCTAGATA<br>ATGAGGAGTGCCACTTGCAAA<br>/56-FAM/CACACGAAA/ZEN/TGTGCCAATGCAAGTCCTT/3IABkFQ/
B2M	AAGTGGGATCGAGACATGTAAG<br>GCAAGCAAGCAGAATTTGGA<br>/56-JOEN/TCATGGAGG/ZEN/TTTGAAGATGCCGCA/3IABkFQ/

4C-seq

Viewpoint (enzyme combo.)	Reading Primer (5'>3')	Non-reading Primer (5'>3')
CFTR -80.1kb (NlaIII/Csp6I)	tacacgacgctcttccgatctACTGAGAACTTACAGGGCAGTC	actggagttcagacgtgtgctcttccgatctCTGGTAGCTTTTGGTTGAATG
CFTR -20.9 kb (NlaIII/DpnII)	tacacgacgctcttccgatctTTAACAAAGTTTAGGTAAATGACCA	actggagttcagacgtgtgctcttccgatctTTAACAAAGTTTAGGTAAATGACCA
CFTR Promoter (NlaIII/Csp6I)	tacacgacgctcttccgatctGCACTTACTATATGCAGGCATG	actggagttcagacgtgtgctcttccgatctTGAAGTGTTCTTTGGATATTGC
CFTR int1+19.5kb (NlaIII/DpnII)	tacacgacgctcttccgatctAGGTGTGTCTGCTTTGTCTC	actggagttcagacgtgtgctcttccgatctGTCCTTTGTTTGTTTGTTTG
CFTR +48.9kb (NlaIII/DpnII)	TACACGACGCTCTTCCGATCTGAGTGAGCTTGAAAGCCATG	actggagttcagacgtgtgctcttccgatctTGGAACATCGTCAGTGGAAG
